# Central and Peripheral Alterations of Retinal and Choroidal Vasculature in Multiple Sclerosis: Insights from Multimodal Imaging

**DOI:** 10.1016/j.xops.2026.101192

**Published:** 2026-04-15

**Authors:** Jamie Mitchell, Gemma McIlwaine, Emma Pead, Kenneth Sloan, Giovanni Ometto, Tom MacGillivray, Stella Hughes, Gavin McDonell, Denise C. Fitzgerald, Jill Moffat, David Wright, Ruth E. Hogg, Tunde Peto, Imre Lengyel, Lajos Csincsik

**Affiliations:** 1 Wellcome Wolfson Institute for Experimental Medicine, Queen’s University Belfast, Belfast, UK; 2 Institute for Neuroscience and Cardiovascular Research, The University of Edinburgh, Edinburgh, UK; 3 Department of Computer Science, University of Alabama at Birmingham, Birmingham, Alabama; 4 Department of Optometry and Visual Sciences, City St George’s University of London, London, UK; 5 Belfast Health and Social Care Trust, The Royal Victoria Hospital, Belfast, UK; 6 Centre for Public Health, Queen’s University Belfast, Belfast, UK

**Keywords:** Multiple sclerosis, Multimodal retinal imaging, Ultra-widefield imaging, Retinal vasculature, Choroidal vasculature

## Abstract

**Purpose:**

To evaluate retinal and choroidal vascular alterations in multiple sclerosis (MS) using multimodal imaging and determine their association with disability and disease progression.

**Design:**

Prospective, observational, single center, cross-sectional, case–control study.

**Participants:**

Sixteen MS and 25 control participants.

**Methods:**

Multimodal retinal imaging, including color fundus photography, ultra-widefield imaging, OCT, and OCT angiography, were performed. Retinal vascular parameters (RVPs) and choroidal vascular parameters (CVPs) were compared between control eyes and eyes with (eyes with a history of optic neuritis [MSON]) and without optic neuritis (ON) (eyes without a history of ON [MSnON]) using regression models. Associations of RVPs and CVPs with Expanded Disability Status Scale (EDSS) scores and annual EDSS progression rate were assessed. Subanalysis compared RVPs and CVPs between MSnON and MSON eyes using Wilcoxon rank-sum tests.

**Main Outcome Measures:**

Differences in RVPs and CVPs between groups and associations with EDSS and annual rate of EDSS progression.

**Results:**

Compared with controls, MSnON eyes showed narrower central retinal venules (*P* = 0.014), increased arteriole-to-venule ratio (AVR) (*P* = 0.031), and reduced venular fractal dimension (FD) (*P* = 0.013). Disability correlated with increased venular caliber (*P* < 0.001), vessel density (*P* = 0.026), superficial vascular complex (SVC) capillary density (*P* < 0.001), and choroidal thickness (*P* = 0.006), and decreased AVR (*P* = 0.006) and choroidal vascularity index (*P* = 0.030). Annual EDSS progression was associated with increased arteriolar caliber (*P* = 0.001), AVR (*P*= 0.025), SVC capillary density (*P* < 0.001), foveal avascular zone (FAZ) volume (*P* < 0.001), and deep FAZ area (*P* < 0.001) and a decreased venular width gradient (*P* = 0.011) and FD (*P* < 0.001). Eyes with a history of ON eyes showed narrower venular caliber (*P* = 0.008), density (*P* = 0.012), and FD (*P* = 0.006). When MSON and MSnON are compared, ON affected only central arteriolar caliber (*P* = 0.010) and global SVC density (*P* = 0.010).

**Conclusions:**

Structural retinal and choroidal vascular alterations in MS were associated with disability and disease progression. These findings highlight the importance of retinal vascular assessments in the diagnosis, monitoring, and prognostication of MS, warranting confirmation in longitudinal studies.

**Financial Disclosure(s):**

Proprietary or commercial disclosure may be found in the Footnotes and Disclosures at the end of this article.

In multiple sclerosis (MS), early diagnosis, effective monitoring of disease progression, and evaluation of treatment response remain critical yet challenging. Neuroimaging has long been the cornerstone of MS assessment; however, its limited resolution and high cost can impede the detection of pathological changes and restrict accessibility in routine clinical practice. Consequently, there is an urgent need for more accessible, cost-effective biomarkers that can capture early or progressive changes in the CNS.

The retina, as an extension of the CNS, provides a noninvasive and high-resolution window into neurodegenerative and vascular processes in the brain. In MS, inner retinal neurodegeneration is well documented,^1^^,^^2^ with emerging evidence of outer retinal involvement.^3^^,^^4^ Retinal vascular alterations also appear to mirror cerebrovascular pathology in MS. Quantitative retinal imaging reveals reduced retinal blood flow velocities and perfusion volumes in individuals with MS compared with controls, providing evidence of impaired microcirculation.^5^ Physiological studies further demonstrate reduced total retinal oxygen extraction and lower retinal blood flow, particularly in eyes with a history of optic neuritis (MSON), linking functional vascular alterations with structural changes.^6^ Functional abnormalities, including increased arteriolar oxygenation and impaired vascular reactivity, parallel cerebral vascular dysfunction.^6^, ^7^, ^8^, ^9^, ^10^ Meta-analyses of OCT angiography (OCTA) indicate lower superficial vascular complex (SVC) density in MS eyes relative to controls, confirming microcapillary involvement at the macular level.^11^^,^^12^ Yet, structural changes to the retinal and choroidal vasculature remain poorly characterized, with most studies limited to microcapillary alterations in the central retina, overlooking changes to the larger vessels (arterioles and venules) as well as the peripheral retina.^11^^,^^12^

Despite these advances, 2 important areas remain underexplored: (1) the potential pathology of larger retinal vessels (arterioles and venules) that integrate upstream vascular tone and downstream metabolic demand and (2) disease signals in the retinal periphery, where inflammatory vasculopathy may manifest (periphlebitis^13^) and where a substantial portion of the vascular tree resides.^14^ These gaps are clinically important: macrovascular remodeling may precede or amplify microcapillary loss by altering local hemodynamics, thereby creating a permissive environment for secondary microvascular injury. Several lines of evidence motivate investigation of these compartments. First, OCT-based analyses around the optic disc (OD) report fewer and narrower peripapillary blood vessels, reduced branching complexity, and associations with disability in MS,^15^ implicating macrovascular remodeling that extends beyond capillary loss. Second, ultra-widefield (UWF) imaging identifies peripheral vessel caliber reductions and structural irregularities not captured by standard fundus photography,^16^ suggesting that disease-related vascular changes extend well beyond the posterior pole. Together, these observations indicate that limiting assessment to the macular microcirculation risks underestimating the full extent of retinal vascular pathology in MS and potentially overlooking biomarkers more closely linked to disability and progression.

Here, we used multimodal retinal imaging—integrating color fundus photography (CFP), UWF imaging, OCT, and OCTA—to enable a more comprehensive assessment encompassing: (1) macrovascular features of arterioles and venules (caliber, tortuosity [TORT], density, fractal dimension [FD]); (2) peripheral vascular organization, including width gradient (WG) and FD over extended fields; and (3) microcapillary metrics, including SVC and deep vascular complex (DVC) density, foveal avascular zone (FAZ) metrics, and choroidal structure (thickness and vascularity index). We evaluated these changes in eyes with and without a history of optic neuritis (ON) from individuals with relapsing-remitting MS in relation to eyes of control participants without MS. By linking vascular parameters to physical disability, we aimed to identify accessible structural biomarkers for potential diagnosis, monitoring, and prognostication in future longitudinal studies and clinical trials.

## Methods

### Study Design and Recruitment

The Belfast Eye and MS (BEAMS) study is a prospective, observational, cross-sectional, case–control study to assess the feasibility and utility of multimodal retinal imaging in MS, which was approved by the United Kingdom Research Ethics Committee (REC 18/NW/0334). Each participant provided informed written consent, and the study followed the Declaration of Helsinki. Participants were recruited through the Northern Ireland MS Research Network via postal invitations, which included a reply slip and a freepost envelope, sent to individuals who had previously agreed to be contacted about future MS studies. Control participants were recruited from the Queen’s University Belfast community, including staff and students, through institutional social media channels and wall posters. Demographic, information on comorbidities, medications/treatments, diabetes, hypertension, coronary artery disease, cerebrovascular disease diagnosis, physical disability, and prior history of ON was obtained through medical records for the MS cohort; in the control cohort, this information was collected through a questionnaire. Physical disability in MS was assessed using the Expanded Disability Status Scale (EDSS), a clinician-administered ordinal scale ranging from 0 (no disability) to 10 (death due to MS), which quantifies neurological impairment across 8 functional systems—including pyramidal, cerebellar, brainstem, sensory, bowel and bladder, visual, cerebral, and other functions—with higher scores reflecting greater disability. The EDSS is the most widely used outcome measure in MS clinical trials and observational studies, and provides a standardized, reproducible measure of functional decline. Expanded Disability Status Scale was recorded from the clinical visit closest to the study date, and the annual rate of disability progression was calculated as the difference between EDSS at the study visit and at diagnosis, divided by the number of years since diagnosis.

All participants underwent a full ophthalmic examination, including best-corrected visual acuity, refractometry (Auto-refractor [Shin Nippon Accuref K-900]) intraocular pressure (IOP) (Reichert Ocular Response Analyzer), dilated fundus examination (slit lamp biomicroscopy), and comprehensive ocular history review.

The exclusion criteria for both cohorts included a history of ON within 6 months of the study visit, any inflammatory condition in controls, and any inflammatory condition other than MS in participants with MS. Additional ocular exclusions comprised glaucoma, retinal vascular disease, a history of traumatic or toxic optic neuropathy, and the use of high-dose corticosteroids. Individuals with significant refractive error—defined as a spherical equivalent worse than ±6.5 diopters—or evidence of pathological myopia were excluded to minimize refractive-related effects on retinal or choroidal measurements. Systemic exclusion criteria also included coronary artery disease and cerebrovascular disease.

### Retinal and Choroidal Imaging

After pharmacologic pupil dilation with 1% w/v tropicamide in both eyes, multimodal imaging was performed bilaterally for all participants. Multimodal retinal imaging included the acquisition of CFPs (Canon CX-1, Canon Inc), UWF images (200DTx, Optos plc), and OCTA scans (Heidelberg Engineering GmbH, Camera Model: S3610). Choroidal imaging included acquisition of OCT scans in enhanced depth imaging (EDI) mode using the same Heidelberg device as for OCTA. As part of the study, retinal vascular parameters (RVPs)—including characteristics of retinal arterioles, venules, and microcapillaries—and choroidal vascular parameters (CVPs) were investigated.

Each imaging modality underwent its own dedicated automated and manual quality control (QC) procedures before data analysis. Only scans that were deemed sufficiently reliable for extracting the specific parameters associated with that modality were included. Because the QC criteria and image quality requirements differ between modalities—or within modality between parameters—a scan may pass QC for one modality but fail for another. As a result, the number of eyes available for analysis can vary across parameter sets. More details on imaging parameters, corresponding QC, and the exact number of eyes included for each modality are provided in the corresponding subsections.

### RVPs—Arterioles and Venules

#### Central Retina

Some vessel characteristics in the central retina (posterior pole) were extracted from OD-centered CFPs using the publicly available deep learning algorithm Automorph.^17^ Automorph is an externally validated deep learning pipeline for automated analysis of retinal vascular morphology on CFPs facilitating widespread research in ophthalmic and systemic diseases. Automorph incorporates a robust, built-in QC system that permits only retinal images deemed sufficiently segmentable and capable of yielding reliable outputs to proceed to analysis.^17^ Retinal vascular parameters were extracted from 2 concentric zones centered on the OD: zone B, extending from 0.5 to 1 OD diameter and zone C, spanning 0.5 to 2 OD diameters from the OD margin (Fig S1/1A, available at www.ophthalmologyscience.org). Vascular measurements included vessel caliber metrics such as central retinal arteriolar equivalent (CRAE), central retinal venular equivalent (CRVE), and arteriolar-to-venular ratio (AVR), derived from zone B using the Knudtson method.^18^ Additional parameters, such as vessel TORT, vessel density (VD), and FD, were extracted from zone C. Vessel density refers to the proportion of pixels identified as vessels relative to the total number of pixels within a given region. Fractal dimension represents the branching complexity of the retinal vascular tree, and TORT is the measure of vessel twisting and bending along its path. After Automorph’s built-in, robust QC step,^17^ this analysis included 8 MSON and 14 eyes without a history of ON (MSnON) from 12 MS and 35 eyes from 19 controls.

#### Peripheral Retina

Retinal vascular parameters from UWF images were extracted by using the Vasculature Assessment Platform for Images of the Retina-UWF (VAMPIRE) software, an application for efficient, semiautomatic quantification of retinal vessel properties, including WG and FD^19^ (Fig S1/1B, C). Images with extensive eyelash or eyelid obstructions, media opacities, or blurred vessels were excluded from analysis if they affected the extended zone.

For WG calculations, the operator manually identified the most prominent, unbroken vessel paths segmented by the software. These were the largest and longest vessels in each quadrant that were not obscured or fragmented by imaging artefacts (Fig S1/1B). The vessels were automatically classified into arterioles and venules by the software and manually corrected by the trained operator (J.M.) if necessary. Width gradient was calculated by the software by measuring vessel width at regular intervals along its path, and the gradient of thinning was fitted using a robust regression line.^20^^,^^21^ After extraction of WG measurements from each quadrant of the image, a global value was calculated by averaging them.

For fractal extraction, a standardized region of interest was previously defined to provide maximum coverage of the retina without eyelids and eyelashes (extended zone, Fig S1/1C) after aligning images using the OD–foveal axis.^22^ This corresponds to an area of approximately 319 mm^2^ used for data extraction. Fractal dimension was calculated by the sandbox count method.^23^ The FD segmentation module of the VAMPIRE software used in this study did not allow vessel classification; therefore, the vascular tree complexity includes both arterioles and venules. After QC, this analysis included 9 MSON, 18 MSnON eyes from 14 MS, and 43 eyes from 22 controls.

### RVPs—Microcapillaries

#### Microcapillary Density

Microcapillary densities were calculated for the SVC and DVCs using 10 × 10 mm OCTA scans (512 B-scans per volume and 512 A-scans per B-scan at high resolution, with an automated real-time of 7), analyzed with the OCTA Analyzer software developed by Heidelberg (SP-X1902). The SVC slab was defined as the region between the inner limiting membrane and the outer boundary of the inner plexiform layer. The DVC slab was defined as the region between the outer boundary of the inner plexiform layer and the outer boundary of the outer plexiform layer (Fig S1/2A). Capillary density values (binary classification) were automatically calculated by the software for both the inner (1–2 mm) and outer rings (ORs) (2–3 mm) of the ETDRS grid, as well as globally after applying built-in filters to remove projection artefacts (3-dimensional [3D] projection artefact removal PAR algorithm) (Fig S1/2B, C).

Quality control for imaging artefacts followed the method published by Hogg et al.^24^ Scans graded as 1 or 2 on a 5-point scale were excluded from analysis to minimize measurement errors due to poor image quality. After QC, this analysis included 6 MSON and 17 MSnON eyes from 12 MS and 46 eyes from 23 controls.

#### FAZ

OCT angiography scans were exported from the Heyex software as individual tagged image file format files, and the FAZ area (mm^2^) and volume (mm^3^) were measured using a previously reported method.^25^ All processing was performed in Matlab R2020a software (MathWorks) using the Image Processing Toolbox. The algorithm identifies the vasculature of 3 networks: superficial capillary complex, intermediate capillary plexus, and deep capillary plexus, each calculated independently by identifying the boundaries between layers. Finally, with the known layers and the inner limiting membrane and outer plexiform layer segmentation, the algorithm calculates the 3D FAZ and FAZ areas for SVC, intermediate capillary plexus, and deep capillary plexus. After QC, this analysis included 7 MSON and 17 MSnON eyes from 14 MS and 40 eyes from 22 controls.

### CVPs

#### Choroidal Thickness and Choroidal Vascularity Index

Thirty × 20 mm OCT volume scans, centered on the fovea, were acquired using EDI mode (Fig S1/3A and B). Each scan consisted of 25 B-scans per volume, spaced 240 μm apart, with each B-scan comprising 1536 A-scans (high resolution) and an automated real-time of 9. If an EDI image was unavailable due to poor quality or was not acquired, a 30 × 25 mm volume scan was used instead, consisting of 61 B-scans spaced 120 μm apart, acquired without EDI. These B-scans were composed of 768 A-scans each (high-speed mode) with an automated real-time of 15.

Choroidal inner boundary (Bruch’s membrane) was segmented automatically using the Heyex software. All automated segmentations were subsequently reviewed for potential errors to ensure that inaccurate boundary detection did not introduce measurement artefacts. OCT scans exhibiting poor signal quality or clipping artefacts that compromised reliable segmentation were excluded from data extraction and analysis.

Scans were exported using Heyex’s XML export function in tagged image file format, accompanied by metadata in XML format containing coordinates of the choroidal inner boundary and information necessary for volumetric scan reconstruction in ImageJ (National Institutes of Health). Choroidal thickness (CT) and choroidal vascularity index (CVI) were quantified for the central circle (1 mm), inner ring (1–3 mm), OR (3–6 mm), and for the entire area (global) of the ETDRS grid, following the manual labeling of choroidal outer boundary by a trained operator (J.M.), who was masked to the eye status. The segmentation of the choroidal outer boundary and the subsequent automated binarization and creation of a 3D CT and CVI map images were carried out using a series of homebuilt ImageJ plugins developed by one of the authors (K.S.) and available via the ImageJ Update Site (https://sites.imagej.net/CreativeComputation/) (Fig S1/3C–E). The inner border of the sclera, known as the choroidal scleral interface, was considered the outer boundary of the choroid. When the suprachoroidal layer—comprising the hyperreflective suprachoroidal stroma and the hyporeflective suprachoroidal space—was present, the inner border of the sclera was located posterior to the suprachoroidal space. In the absence of the suprachoroidal layer, the inner border of the sclera corresponded to the interface between the hyporeflective vasculature and the hyperreflective sclera.^26^

The automated binarization and 3D CVI map creation steps involved Gaussian smoothing (sigma = 0.5 pixels), conversion to an 8-bit binary image, Otsu autolocal thresholding, and erosion and dilation to avoid fragmentation (remove too small regions). White pixels were accepted as the stromal area, and the dark pixels were accepted as the luminal area, corresponding to the blood vessels (Fig S1/3C). This was done at multiple scales to capture a wide range of vessel sizes. The CVI, which was the proportion of the luminal area to the total choroidal area, was calculated. After QC, this analysis included 10 MSON, 20 MSnON eyes of 15 MS, and 47 eyes of 24 control.

### Statistical Analysis

RStudio (2024.04.2+764, R Foundation for Statistical Computing) with R version 4.3.2 (2023-10-31) was used for data visualization and statistical analyses. Cohort characteristics were summarized using descriptive statistics. Categorical variables were reported as absolute frequencies and percentages, and continuous variables as mean values with standard deviation, median value with interquartile range, and range (minimum and maximum values). To visually assess the distribution of vascular parameters, box plots were generated. Group differences were evaluated using Pearson chi-square test and Fisher exact test for categorical variables, and the Wilcoxon or Kruskal–Wallis rank-sum tests for continuous variables.

We employed generalized estimating equations, linear regression, univariate (unadjusted), and multivariate (adjusted) models to evaluate the relationship between vascular changes (outcome) and diagnosis (exposure). The exposure variable included 3 levels: MSON, MSnON, and controls, with control eyes serving as the reference group. This analysis accounted for the correlation between eyes within the same patient using an exchangeable correlation matrix and, in the multivariate models, included adjustments for relevant confounders as covariates. Covariates in the adjusted model were limited to those variables that showed a significant association with the exposure, which included IOP and hypertension.

To evaluate the associations between disability measures (EDSS and annual EDSS progression) and retinal or CVPs, we performed generalized estimating equation linear regression analyses restricted to MSnON eyes. Equivalent models could not be generated for the MSON subgroup due to the small sample size. These models were adjusted for age, sex, refractive error, and hypertension, as these factors can influence vascular measurements.

To explore the effect of ON history on RVPs and CVPs, we conducted a subanalysis comparing MSON eyes to MSnON eyes using the Wilcoxon rank-sum test, and the results are presented in the supplementary material. The significance level was evaluated at a false discovery rate-adjusted alpha = 0.05 to account for multiple comparisons.

## Results

The study cohort consisted of 16 participants with MS and 25 controls. Of the 32 eyes of individuals with MS, 21 (65.6%) had no history of ON (MSnON) while 11 (34.4%) had experienced a past event of ON (MSON). There were no significant group differences in age (*P* = 0.309), sex (*P* = 0.365), or spherical refractive error (*P* = 0.063) (Table 1). However, both the MSON and MSnON groups demonstrated lower IOP values (*P* = 0.004) and a greater prevalence of hypertension (*P* < 0.001) compared with the control group (Table 1).

**Table 1:** Characteristics of Study Participants

	MSON	MSnON	Ctrl	*P* Value∗
Cases	N = 16		N = 25	
Eyes	n = 11	n = 21	n = 50	
Age				0.309
Mean (SD)	56 (10)	51 (11)	48 (12)	
Median (IQR)	55 (47, 63)	51 (41, 59)	52 (38, 57)	
Range	44, 73	34, 73	28, 71	
Sex				0.365
Male	4 (50%)	7 (53.8%)	8 (32%)	
Female	4 (50%)	6 (46.2%)	17 (68%)	
IOPg				**0.004**
Mean (SD)	13.1 (3.1)	13.0 (3.4)	15.7 (2.9)	
Median (IQR)	11.6 (11.1, 15.4)	12.7 (11.3, 15.7)	15.8 (14.0, 17.5)	
Range	8.6, 18.6	7.3, 18.8	10.1, 22.6	
Spherical error				0.063
Mean (SD)	1.69 (3.15)	0.75 (1.24)	–0.25 (1.82)	
Median (IQR)	1.25 (–0.50, 4.75)	0.50 (–0.12, 1.62)	0.00 (–0.75, 0.75)	
Range	–2.75, 6.25	–0.75, 3.75	–5.87, 4.62	
Hypertension				**<0.001**
NO	2 (25%)	7 (53.8%)	23 (92.0%)	
YES	6 (75%)	6 (46.2%)	2 (8.0%)	
MS subtype				
RRMS	6 (75%)	10 (76.9%)	N/A	>0.999
SPMS	2 (25%)	3 (23.1%)	N/A	

Ctrl = control; IOPg = intraocular pressure (Goldmann); IQR = interquartile range; MSnON = eyes with no prior history of optic neuritis; MSON = eyes with prior history of optic neuritis; N/A = not applicable; RRMS = relapsing-remitting multiple sclerosis; SD = standard deviation; SPMS = secondary progressive multiple sclerosis.

Summary of demographic and clinical characteristics of study participants, including healthy controls and individuals with multiple sclerosis.

Statistically significant values are indicated in bold.

∗ Chi-square test and Kruskal–Wallis rank-sum test.

Among the MS participants, 13 had relapsing-remitting MS and 3 had secondary progressive MS (Table 2). All but 5 were receiving disease-modifying therapies. The mean disease duration since diagnosis was 13 ± 9 years (range: 3–34 years). The mean EDSS score was 3.44 ± 2.33 (range: 1.00–8.00), with a mean annual progression of 0.21 ± 0.16 (range: 0.04–0.60) (Table 2). Summary tables and plots for all vascular parameters are provided in the supplementary document (Tables S1–3; Figs S2–S8, available at www.ophthalmologyscience.org).

**Table 2:** Clinical Characteristics of the MS Cohort

Number of Cases	MS Characteristics		MS Characteristics by Subtype		
	N	N = 16∗	N	RRMS N = 13∗	SPMS N = 3∗
Age	16		16		
Mean (SD)		51 (11)		47 (8)	66 (7)
Median (IQR)		50 (43, 59)		46 (41, 53)	65 (59, 73)
Min, max		34, 73		34, 60	59, 73
Treated	16		16		
NO		5 (31.3%)		3 (23.1%)	2 (66.7%)
YES		11 (68.8%)		10 (76.9%)	1 (33.3%)
Treatment type	16		16		
Untreated		5 (31.3%)		3 (23.1%)	2 (66.7%)
Interferon		2 (12.5%)		1 (7.7%)	1 (33.3%)
Glatiramer acetate		1 (6.3%)		1 (7.7%)	0 (0.0%)
Dimethyl fumarate		1 (6.3%)		1 (7.7%)	0 (0.0%)
Natalizumab		7 (43.8%)		7 (53.8%)	0 (0.0%)
Disease duration (y)	16		16		
Mean (SD)		13 (9)		11 (9)	20 (2)
Median (IQR)		10 (6, 19)		8 (5, 14)	21 (18, 22)
Min, max		3, 34		3, 34	18, 22
EDSS	16		16		
Mean (SD)		3.44 (2.34)		2.58 (1.54)	7.17 (1.04)
Median (IQR)		2.75 (1.50, 5.25)		2.50 (1.00, 3.00)	7.50 (6.00, 8.00)
Min, max		1.00, 8.00		1.00, 6.00	6.00, 8.00
EDSS (progression/yr)	16		16		
Mean (SD)		0.41 (0.47)		0.43 (0.52)	0.36 (0.09)
Median (IQR)		0.30 (0.13, 0.48)		0.20 (0.11, 0.50)	0.36 (0.27, 0.44)
Min, max		0.07, 2.00		0.07, 2.00	0.27, 0.44

EDSS = Expanded Disability Status Scale; IQR = interquartile range; max = maximum; min = minimum; MS = multiple sclerosis; RRMS = relapsing-remitting multiple sclerosis; SD = standard deviation; SPMS = secondary progressive multiple sclerosis.

This table presents clinical characteristics of the MS cohort, stratified by MS subtype: relapsing-remitting MS and secondary progressive MS.

∗ n (%).

### RVP and CVP Differences between MS and Controls

When assessing vessel caliber metrics from CFPs, we observed a reduction in CRVE in both MSnON (β = –2.591; *P* = 0.014) and MSON eyes (β = –3.466; *P* = 0.008) compared with control eyes in the multivariable-adjusted model. A decrease in AVR was also detected in MSnON eyes (β = –0.073; *P* = 0.031) (Table 3). When UWF imaging was used to assess vascular tapering toward the retinal periphery (WG), no significant differences were detected between MS and control eyes in the final adjusted model (Table 3).

**Table 3:** Retinal Vascular Parameters of Retinal Arterioles and Venules in MSON and MSnON vs. Ctrl Eyes

RVPs/Location	Modality	Location	GEE – Unadjusted MSnON			GEE – Unadjusted MSON			GEE – Adjusted MSnON			GEE – Adjusted MSON		
			Beta	95% CI	*P* Value	Beta	95% CI	*P* Value	Beta	95% CI	*P* Value∗	Beta	95% CI	*P* Value∗
Vessel caliber measures														
CRAE (px)	CFP	Zone B	0.608	–1.490 to 2.706	0.570	–2.821	–5.009 to –0.633	**0.023**	1.144	–1.227 to 3.516	0.509	–2.094	–4.859 to 0.671	0.509
CRVE (px)	CFP	Zone B	–1.428	–3.611 to 0.756	0.200	–1.623	–4.023 to 0.776	0.200	–2.591	–4.571 to –0.612	**0.014**	–3.466	–5.817 to –1.116	**0.008**
AVR	CFP	Zone B	0.042	–0.013 to 0.097	0.162	–0.042	–0.101 to 0.017	0.162	0.073	0.019 to 0.128	**0.031**	0.007	–0.062 to 0.077	0.842
WGa (μm/mm)	UWF	Entire image	–0.097	–0.625 to 0.430	0.847	0.046	–0.417 to 0.508	0.847	0.225	–0.354 to 0.804	0.447	0.460	–0.098 to 1.019	0.142
WGv (μm/mm)	UWF	Entire image	–0.543	–0.970 to –0.116	**0.025**	–0.172	–0.791 to 0.446	0.585	–0.170	–0.577 to 0.236	0.411	0.506	–0.050 to 1.062	0.148
Vessel tortuosity														
TORT	CFP	Zone C	–0.023	–0.045 to 0.000	0.099	–0.005	–0.044 to 0.034	0.801	–0.024	–0.050 to 0.002	0.140	0.011	–0.019 to 0.040	0.050
TORTa	CFP	Zone C	–0.014	–0.056 to 0.028	0.629	0.015	–0.047 to 0.078	0.629	0.011	–0.036 to 0.059	0.647	0.050	–0.003 to 0.102	0.086
TORTv	CFP	Zone C	–0.016	–0.042 to 0.011	0.344	0.019	–0.020 to 0.059	0.344	0.001	–0.031 to 0.029	0.983	0.031	–0.007 to 0.069	0.228
Vessel density														
VD	CFP	Zone C	0.0002	–0.003 to 0.006	0.460	–0.003	–0.008 to 0.003	0.460	0.001	–0.005 to 0.007	0.825	–0.006	–0.012 to 0.000	0.095
VDa	CFP	Zone C	0.001	–0.001 to 0.004	0.261	–0.002	–0.005 to 0.001	0.261	0.001	–0.002 to 0.004	0.819	–0.004	–0.007 to 0.000	0.062
VDv	CFP	Zone C	0.000	–0.003 to 0.003	0.920	–0.002	–0.004 to 0.001	0.449	–0.002	–0.005 to 0.002	0.483	–0.004	–0.007 to –0.001	**0.012**
Vascular tree complexity														
FD	CFP	Zone C	0.000	–0.020 to 0.020	0.991	–0.019	–0.047 to 0.010	0.386	–0.008	–0.033 to 0.017	0.528	–0.033	–0.064 to –0.003	0.128
FDa	CFP	Zone C	0.008	–0.012 to 0.027	0.451	–0.022	–0.057 to 0.012	0.418	0.003	–0.024 to 0.031	0.963	–0.037	–0.071 to –0.002	0.078
FDv	CFP	Zone C	–0.018	–0.040 to 0.004	0.109	–0.020	–0.042 to 0.002	–0.020	–0.034	–0.061 to –0.008	**0.013**	–0.041	–0.066 to –0.016	**0.006**
FD	UWF	Extended zone	0.000	–0.022 to 0.022	0.992	–0.001	–0.024 to 0.022	0.992	0.012	–0.009 to 0.032	0.257	0.017	–0.005 to 0.040	0.174

a = arteriole; AVR = arteriole-to-venule ratio; CFP = color fundus photography; CI = confidence interval; Ctrl = control; CRAE = central retinal arteriolar equivalent; CRVE = central retinal venular equivalent; FD = fractal dimension; GEE = generalized estimating equation; MSnON = eyes with no prior history of optic neuritis; MSON = eyes with prior history of optic neuritis; RVP = retinal vascular parameter; TORT = tortuosity; UWF = ultra-widefield; v = venule; VD = vessel density; WG = width gradient; WGv = venular width gradient.

All *P* values are adjusted for multiple comparisons using false discovery rate correction.

This table presents the results of linear regression analysis using GEEs, with RVPs as the outcome and diagnosis as the exposure. The beta coefficient represents the mean difference in each vascular parameter between eyes from individuals with multiple sclerosis with and without a history of optic neuritis and healthy controls, with Ctrl serving as the reference group. Statistically significant values are indicated in bold.

∗ The model is adjusted for intraocular pressure and hypertension.

Analysis of central retinal vascular geometry demonstrated a significant reduction in venular VD in MSON eyes relative to controls (β = –0.004; *P* = 0.012) (Table 3). Examination of retinal vascular tree complexity showed lower venular FD values in both MSnON (β = –0.034; *P* = 0.013) and MSON eyes (β = –0.041; *P* = 0.006) compared with controls in the multivariable model (Table 3). When this analysis was extended to the peripheral retina, no significant differences were observed between MS and control eyes (Table 3).

Analysis of microcapillary network using OCTA revealed a significant reduction in SVC density in MSON eyes compared with controls (β = –3.851; *P* = 0.001) (Table 4). No differences were observed in FAZ size or area between MS and control eyes (Table 4).

**Table 4:** Retinal Vascular Parameters of Microcapillaries in MSON and MSnON vs. Ctrl Eyes

RVPs/Location	Modality	GEE – Unadjusted MSnON			GEE – Unadjusted MSON			GEE – Adjusted MSnON			GEE – Adjusted MSON		
		Beta	95% CI	*P* Value	Beta	95% CI	*P* Value	Beta	95% CI	*P* Value∗	Beta	95% CI	*P* Value∗
SVC capillary density													
Inner ring	OCTA	–0.643	–2.797 to 1.511	0.558	–4.360	–7.647 to –1.073	**0.019**	0.455	–2.252 to 3.163	0.898	–2.720	–6.143 to 0.704	0.239
Outer ring	OCTA	–276	–2.281 to 1.729	0.787	–3.196	–5.543 to –0.849	**0.015**	0.235	–2.408 to 2.877	0.932	–2.455	–5.879 to 0.969	0.640
Global	OCTA	–0.416	–2.115 to 1.283	0.632	–3.851	–6.019 to –1.682	**0.001**	0.407	–1.958 to 2.771	0.845	–2.639	–5.496 to 0.218	0.243
DVC capillary density													
Inner ring	OCTA	1.301	–0.801 to 3.402	0.450	0.835	–1.462 to 3.133	0.476	1.338	–1.097 to 3.772	0.509	1.274	–1.609 to 4.156	0.509
Outer ring	OCTA	2.798	0.647 to 4.949	**0.022**	2.906	–0.676 to 6.488	0.112	2.116	–0.488 to 4.719	0.445	2.251	–2.116 to 6.619	0.491
Global	OCTA	2.022	–0.042 to 4.086	0.110	1.907	–0.841 to 4.654	0.174	1.708	–0.749 to 4.164	0.542	1.809	–1.686 to 5.303	0.542
FAZ													
FAZ volume (mm3)	OCTA	–0.001	–0.005 to 0.003	0.941	0.000	–0.006 to 0.006	0.941	–0.001	–0.005 to 0.004	0.9757	0.000	–0.005 to 0.005	0.974
SUP FAZ area (mm^2^)	OCTA	0.014	–0.116 to 0.143	0.833	0.142	–0.050 to 0.335	0.292	0.003	–0.139 to 0.145	0.968	0.106	–0.101 to 0.314	0.631
INT FAZ area (mm^2^)	OCTA	0.000	–0.074 to 0.075	0.991	0.045	–0.056 to 0.146	0.768	–0.006	–0.092 to 0.079	0.886	0.025	–0.072 to 0.122	0.816
Deep FAZ area (mm^2^)	OCTA	0.020	–0.077 to 0.118	0.683	0.170	0.022 to 0.318	**0.049**	0.018	–0.092 to 0.127	0.752	0.157	–0.003 to 0.316	0.215

CI = confidence interval; Ctrl = control; DVC = deep vascular complex; FAZ = foveal avascular zone; GEE = generalized estimating equation; INT = intermediate; MSnON = multiple sclerosis with no history of optic neuritis; MSON = multiple sclerosis with a history of optic neuritis; OCTA = OCT angiography; RVP = retinal vascular parameter; SUP = superior; SVC = superficial vascular complex.

All *P* values are adjusted for multiple comparisons using false discovery rate correction.

This table presents the results of linear regression analysis using GEEs, with retinal microcapillary density as the outcome and diagnosis as the exposure. The beta coefficient represents the mean change in each microcapillary parameter between eyes from individuals with multiple sclerosis with and without a history of optic neuritis and healthy controls, with Ctrl serving as the reference group. Statistically significant values are indicated in bold.

∗ The model is adjusted for intraocular pressure and hypertension.

Evaluation of CVPs demonstrated no significant differences in CT between MS and control eyes in the adjusted model (Table 5). However, CVI reduced within the central circle of the ETDRS grid in MSON eyes compared with controls (β = –10.04; *P* = 0.049) (Table 5).

**Table 5:** Choroidal Vascular Parameters in MSON and MSnON vs. Ctrl Eyes

CVPs/Location	Modality	GEE – Unadjusted MSnON			GEE – Unadjusted MSON			GEE – Adjusted MSnON			GEE – Adjusted MSON		
		Beta	95% CI	*P* Value	Beta	95% CI	*P* Value	Beta	95% CI	*P* Value∗	Beta	95% CI	*P* Value∗
CT (μm)													
Central circle	OCT-EDI	13.35	–0.863 to 27.55	0.066	21.10	4.767 to 37.43	**0.023**	8.973	–11.67 to 29.62	0.526	16.71	–6.088 to 39.51	0.526
Inner ring	OCT-EDI	14.77	0.379 to 29.16	**0.044**	20.09	2.487 to 37.69	**0.044**	11.87	–7.779 to 31.52	0.473	17.13	–6.103 to 40.37	0.473
Outer ring	OCT-EDI	15.04	1.818 to 28.25	0.052	14.45	–0.987 to 29.88	0.067	14.45	–2.202 to 31.11	0.356	13.89	–6.483 to 34.27	0.363
Global	OCT-EDI	14.95	1.507 to 28.39	**0.048**	15.87	0.142 to 31.60	**0.048**	13.70	–3.624 to 31.02	0.338	14.65	–6.233 to 35.53	0.338
CVI (%)													
Central circle	OCT-EDI	–1.219	–6.088 to 3.650	0.624	–10.04	–18.80 to –1.282	**0.049**	1.027	–3.683 to 5.737	0.669	–7.382	–14.10 to –0.662	0.125
Inner ring	OCT-EDI	–1.564	–6.591 to 3.464	0.542	–8.454	–17.01 to 0.101	0.106	0.071	–4.681 to 4.822	0.977	–6.492	–12.58 to –0.404	0.147
Outer ring	OCT-EDI	–2.745	–7.206 to 1.715	0.228	–5.404	–13.02 to 2.211	0.228	–1.637	–4.987 to 1.713	0.587	–3.892	–8.572 to 0.787	0.412
Global	OCT-EDI	–2.428	–6.963 to 2.107	0.294	–6.209	–14.02 to 1.604	0.239	–1.171	–4.729 to 2.388	0.692	–4.587	–9.557 to 0.384	0.282

CI = confidence interval; CT = choroidal thickness; Ctrl = control; CVI = choroidal vascularity index; CVP = choroidal vascular parameter; EDI = enhanced depth imaging; GEE = generalized estimating equation; MSnON = eyes with no prior history of optic neuritis; MSON = eyes with prior history of optic neuritis.

All *P* values are adjusted for multiple comparisons using false discovery rate correction.

This table presents the results of linear regression analysis using GEEs, with CVPs as the outcome and diagnosis as the exposure. The beta coefficient represents the mean change in CT and CVI between eyes from individuals with multiple sclerosis with and without a history optic neuritis and healthy controls, with Ctrl serving as the reference group. Statistically significant values are indicated in bold.

∗ The model is adjusted for IOP and hypertension.

### Associations of Disability with RVPs and CVPs

Higher EDSS scores were linked to lower AVR (β: –0.038; *P* = 0.006) and CVI (β: –2.232; *P* = 0.030), but to greater CRVE (β: 0.925; *P* < 0.001), VD (β: 0.006; *P* = 0.026), SVC (β: 1.267; *P* < 0.001), and CT (β: 4.497; *P* = 0.006) (Table 6, Table 7, Table 8).

**Table 6:** Associations between Disability and Retinal Vascular of Arterioles and Venules in MSnON Eyes

	Modality/Location	EDSS MSnON			EDSS Progression/Year MSnON		
		Beta	95% CI	*P* Value^a^	Beta	95% CI	*P* Value^a^
Vessel caliber measures							
CRAE (px)	CFP/zone B	–0.802	–1.798 to 0.195	0.115	9.825	4.497 to 15.15	**0.001**
CRVE (px)	CFP/zone B	0.925	0.474 to 1.375	**<0.001**	2.3	–3.524 to 8.124	0.452
AVR	CFP/zone B	–0.038	–0.060 to –0.015	**0.006**	0.120	0.021 to 0.220	**0.025**
WGa (um/mm)	UWF/entire image	0.259	–0.047 to 0.566	0.481	–0.135	–0.523 to 0.253	0.805
WGv (um/mm)	UWF/entire image	0-0.011	–0.129 to 0.107	0.920	–0.414	–0.713 to –0.116	**0.011**
Vessel tortuosity							
TORT	CFP/zone C	–0.035	–0.073 to 0.004	0.189	–0.265	–0.541 to –0.010	0.148
TORTa	CFP/zone C	–0.020	–0.046 to 0.006	0.337	–0.147	–0.496 to –0.203	0.510
TORTv	CFP/zone C	–0.015	–0.042 to 0.013	0.641	–0.184	–4.034 to 3.666	0.995
Vessel density							
VD	CFP/zone C	0.006	0.002 to 0.010	**0.026**	–0.037	–0.042 to 0.115	0.732
VDa	CFP/zone C	0.002	0.000–0.004	0.079	0.003	0.007–0.013	0.504
VDv	CFP/zone C	0.001	0.000–0.002	0.068	0.009	–0.026 to 0.045	0.840
Vascular tree complexity							
FD	CFP/zone C	0.002	–0.004 to 0.009	0.620	0.036	0.043–0.115	0.468
FDa	CFP/zone C	–0.002	–0.010 to 0.006	0.577	0.005	–0.094 to 0.103	0.928
FDv	CFP/zone C	0.014	–0.005 to 0.032	0.253	0.051	0.032–0.134	0.286
FD	UWF extended	0.001	–0.003 to 0.005	0.950	–0.018	–0.025 to –0.011	**<0.001**

a = arteriole; AVR = arteriole-to-venule ratio; CFP = color fundus photography; CI = confidence interval; CRAE = central retinal arteriolar equivalent; CRVE = central retinal venular equivalent; EDSS = Expanded Disability Status Scale; FD = fractal dimension; MSnON = eyes with no prior history of optic neuritis; TORT = tortuosity; UWF = ultra-widefield; v = venule; VD = vessel density; WG = width gradient; WGv = venular width gradient.

All *P* values are adjusted for multiple comparisons using false discovery rate correction.

This table presents the results of linear regression analysis using generalized estimating equations, examining associations between EDSS scores and retinal vascular parameters in eyes from individuals with multiple sclerosis without history of optic neuritis. The beta coefficient represents the mean change in each vascular parameter per unit increase in EDSS. Statistically significant values are indicated in bold.

a The model is adjusted for age, sex, spherical error, and hypertension.

**Table 7:** Associations between Disability and RVPs of Microcapillaries in MSnON Eyes

	EDSS MSnON			EDSS Progression/Year MSnON		
	Beta	95% CI	*P* Value∗	Beta	95% CI	*P* Value∗
SVC capillary density						
Inner ring	1.167	–0.005 to 2.340	0.135	0.549	–3.768 to 4.867	0.811
Outer ring	1.547	1.126 to 1.967	**<0.001**	3.783	2.330 to 5.236	**<0.001**
Global	1.267	0.652 to 1.881	**<0.001**	1.828	–0.994 to 4.650	0.340
DVC capillary density						
Inner ring	–0.263	–2.577 to 2.050	0.995	–2.815	–4.993 to –0.638	0.056
Outer ring	1.064	–0.942 to 3.069	0.362	–1.084	–3.504 to 1.336	0.627
Global	0.294	–1.761 to 2.349	0.779	–2.060	–4.052 to –0.068	0.214
FAZ						
FAZ volume (mm^3^)	0.000	–0.003 to 0.002	0.849	0.004	0.002 to 0.005	**<0.001**
SUP FAZ area (μm)	0.019	–0.080 to 0.117	0.811	0.153	–0.058 to 0.364	0.348
INT FAZ area (μm)	–0.028	–0.068 to 0.012	0.431	–0.250	–0.766 to 0.267	0.535
Deep FAZ area (μm)	0.034	–0.004 to 0.072	0.136	0.170	0.110 to 0.230	**<0.001**

CI = confidence interval; DVC = deep vascular complex; EDSS = Expanded Disability Status Scale; FAZ = foveal avascular zone; INT = intermediate; MSnON = eyes with no prior history of optic neuritis; RVP = retinal vascular parameter; SUP = superior; SVC = superficial vascular complex.

All *P* values are adjusted for multiple comparisons using false discovery rate correction.

This table presents the results of linear regression analysis using generalized estimating equations, examining associations between EDSS scores and retinal microcapillary parameters in eyes from individuals with multiple sclerosis without history of optic neuritis. The beta coefficient represents the mean change in each vascular parameter per unit increase in EDSS. Parameters include the SVC and DVC measured from the inner ring, outer ring, and globally on the ETDRS grid, the FAZ volume and the SUP, INT, and deep FAZ area. Statistically significant values are indicated in bold.

∗ The model is adjusted for age, sex, spherical error, and hypertension.

**Table 8:** Associations between Disability and Choroidal Vascular Parameters in MSnON Eyes

	EDSS MSnON			EDSS Progression/Year MSnON		
	Beta	95% CI	*P* Value∗	Beta	95% CI	*P* Value∗
CT (μm)						
Central circle	4.644	1.857 to 7.432	**0.001**	1.851	–8.954 to 12.66	0.737
Inner ring	4.554	1.614 to 7.494	**0.003**	–0.928	–13.69 to 11.84	0.887
Outer ring	4.587	1.448 to 7.726	**0.005**	–3.141	–21.38 to 14.55	0.710
Global	4.497	1.400 to 7.594	**0.006**	–2.887	–19.88 to 14.11	0.739
CVI (%)						
Central circle	–1.578	–3.384 to 0.227	0.217	5.717	–2.353 to 13.78	0.340
Inner ring	–2.301	–4.272 to –0.329	0.055	3.533	–6.373 to 13.44	0.485
Outer ring	–2.236	–3.936 to –0.536	**0.025**	2.378	–4.955 to 9.712	0.583
Global	–2.232	–3.976 to –0.487	**0.030**	2.792	–5.095 to 10.68	0.596

CI = confidence interval; CT = choroidal thickness; CVI = choroidal vascularity index; CVP = choroidal vascular parameter; EDSS = Expanded Disability Status Scale; MSnON = eyes with no prior history of optic neuritis.

All *P* values are adjusted for multiple comparisons using false discovery rate correction.

This table presents the results of linear regression analysis using generalized estimating equations, examining associations between EDSS scores and CVPs in eyes from individuals with multiple sclerosis without history of optic neuritis. The beta coefficient represents the mean change in CT andCVI per unit increase in EDSS. Parameters include CT and CVI in the central circle, inner ring, outer ring, and globally on the ETDRS grid. Statistically significant values are indicated in bold.

∗ The model is adjusted for age, sex, spherical error, and hypertension.

Annual EDSS progression was related to reduced venular WG and peripheral FD (β: –0.018; *P* < 0.001), alongside increased CRAE (β: 9.825; *P* = 0.001), AVR (β: 0.120; *P* = 0.025), SVC (β: 3.783; *P* < 0.001), FAZ volume (β: 0.004; *P* < 0.001), and deep FAZ area (β: 0.170; *P* < 0.001) (Tables 6 and 7).

### The Effect of ON on RVPs and CVPs

When vessel caliber-related parameters were examined, CRAE appeared to be affected by a history of ON, with reduced arterial caliber observed in the MSON group compared with MSnON (CRAE: *P* = 0.010) (Table S4, available at www.ophthalmologyscience.org). None of the other vessel caliber parameters (CRVE, AVR, WG) showed significant differences based on ON history (Table S4). Similarly, no significant effect of ON was observed on other RVPs of retinal arterioles and venules—including TORT, VD, or FD—regardless of whether measurements were taken from zone C or the extended zone/entire image (Tables S5 and S6, available at www.ophthalmologyscience.org).

There was a significant impact of ON on SVC, showing reduced capillary density across the ETDRS grid rings (*P* < 0.030) (Table S7, available at www.ophthalmologyscience.org). No significant effect of ON on FAZ volume or any of the FAZ areas (superficial, intermediate, and deep) was detected (Table S7).

No significant effect of ON history was observed when CT or CVI was compared between MSON and MSnON across the rings of the ETDRS grid or globally (Table S8, available at www.ophthalmologyscience.org).

## Discussion

Beyond the hallmark demyelinating lesions characteristic of MS, a growing body of evidence highlights a range of vascular abnormalities within the brain. These include alterations in cerebral blood flow, vessel structure, and compromised integrity of the blood–brain barrier.^27^ Such disruptions may impair the delivery of oxygen and nutrients, potentially serving not only as downstream effects of neuronal injury but also as active contributors to disease progression and clinical severity.

Functional abnormalities in the retina, such as increased arteriolar oxygenation and impaired vascular reactivity, have been observed and appear to parallel cerebral vascular dysfunction.^6^, ^7^, ^8^, ^9^, ^10^ In contrast, structural changes in the retinal and choroidal vasculature remain poorly characterized. Most existing studies focus on the central microcapillary network often overlooking alterations in retinal arterioles and venules, and the peripheral retina.^11^^,^^12^

In this multimodal imaging study of the BEAMS study cohort, we demonstrated that both central and peripheral retinal vascular alterations, along with choroidal changes, are present in MS and occur largely independent of prior ON. These findings underscore the potential of retinal and choroidal vascular features as biomarkers for diagnosis, disease monitoring, and prognostication in MS.

## Retinal Arteriolar and Venular Changes

In MSnON, we observed a reduction in venular caliber and a decrease in the complexity of the venular vascular tree (FD) compared with controls, even after adjusting for hypertension. In contrast, eyes with ON history showed a reduction in arteriolar width. Similar narrowing of retinal vessels has been reported both around the OD^15^ and in the retinal periphery.^16^ The direction and magnitude of caliber changes differed between MSnON and MSON eyes in our cohort—with venular narrowing predominating in MSnON and arteriolar narrowing in MSON—highlighting that vessel-type and ON history are important modifiers of structural remodeling in MS. The functional hemodynamic consequences of these structural changes, including any effects on retinal blood flow velocity, were not assessed in the present study and warrant investigation in future work.

Reductions in FD have been documented in the brains of individuals with MS, even in the absence of visible white matter lesions on MRI,^28^ suggesting that retina imaging may serve as a surrogate marker for cerebral microvascular changes. Although we are not aware of prior studies specifically examining retinal FD in MS, Bhaduri et al^15^ reported a reduced number of vessels around the OD using optic nerve head OCT scans, which may contribute to the lower FD observed in our study.

Interestingly, although MSnON eyes generally exhibited narrower venules and a less complex venular network compared with controls, higher disability scores and faster annual disability progression (EDSS/year) were associated with wider venular caliber within the MS group. This apparent paradox—narrowing at the group level yet dilation with increasing disability within MS—may reflect distinct pathophysiological processes operating at different disease stages or severity strata. This association remained significant after adjusting for age, sex, spherical error, and hypertension. These findings are consistent with observations in the cerebral vasculature, where enlarged bridging veins have been reported in more severely affected patients with MS.^29^ However, we acknowledge that cross-sectional data cannot establish the temporal sequence of these changes, and the association between venular caliber and disability requires confirmation in longitudinal cohorts.

Our analysis of the peripheral retina revealed a vascular attenuation reflected by lower venular WG and reduced FD in the extended zone among individuals with faster annual disability progression. To our knowledge, peripheral FD has not previously been investigated in MS. While Pearson et al^16^ reported reduced vessel caliber in the peripheral retina, they found no correlation with clinical outcomes over a 2-year follow-up. Ortiz-Pérez et al^13^ demonstrated that peripheral retinal periphlebitis was associated with disability progression, raising the possibility that the peripheral vascular changes observed in our study could reflect a shared susceptibility of the peripheral retinal vasculature to MS-related injury. However, as inflammatory activity was not directly assessed in the present study, the mechanistic basis of the peripheral vascular attenuation cannot be established from these data alone, and this hypothesis requires evaluation in future studies with appropriate inflammatory markers or imaging modalities.

## Retinal Microcapillary Changes

Numerous studies have used OCTA to assess retinal microcapillary density in MS. In our cohort, SVC and DVC densities did not differ significantly between MSnON eyes and controls. However, when comparing MSON eyes with controls or with MSnON eyes, we observed lower SVC density in MSON, consistent with the findings of Kallab et al,^6^ who used the same imaging device, microcapillary density extraction methods, and a comparable sample size. A larger sample size may detect microcapillary density differences between MSnON and controls, as suggested by Murphy et al,^30^ who used the same imaging and analysis methodology. This interpretation is further supported by the meta-analyses of Liu et al^11^ and Mohammadi et al,^12^ which included OCTA studies using diverse imaging protocols and data extraction techniques. However, the comparability of findings across such heterogeneous studies remains an open question for future research.

Our finding of no significant differences in DVC density between MS and control eyes aligns with previous reports.^11^^,^^12^ Similarly, we observed no group differences in FAZ area, consistent with Yilmaz et al,^31^ except for a modest increase in deep FAZ area in MSON eyes compared with controls. Notably, FAZ volume extraction is a relatively novel approach, and to our knowledge, no prior studies that have applied this metric in MS.^25^

Interestingly, we found that a faster annual rate of EDSS progression was associated with a denser microcapillary network in the OR of the SVC in MSnON eyes. To our knowledge, this association has not been reported in previous studies. While earlier studies have shown an inverse correlation between EDSS and superficial vascular plexus density,^30^ our findings indicate a positive correlation, independent of age, sex, refractive error, and hypertension.

## Choroidal Vascular Changes

Our analysis did not reveal significant differences in CT or CVI between MSnON eyes and controls. These CT findings are consistent with several previous studies that also reported no significant differences between the MS and control groups.^32^, ^33^, ^34^ In contrast, other studies have reported regional changes in CT: Temel et al^35^ and Esen et al^36^ found reduced subfoveal CT, while Garcia-Martin et al^37^ observed CT around the OD, with effect sizes decreasing progressively with distance from the disc margin. These findings suggest that specific choroidal regions may be more susceptible to MS-related changes, a hypothesis warranting further investigation. In our cohort, we found a significant increase in CT in MSON eyes when compared with controls. However, CT did not differ significantly when comparing MSON eyes with MSnON eyes.

Reduced CVI has been reported by several studies,^33^^,^^35^^,^^38^ potentially reflecting vascular disorganization that may contribute to MS pathophysiology. In our cohort, we observed only a slight decrease of CVI in MSON eyes. When examining the relationship between CVPs and disability measures, we found that CT positively correlates with EDSS, while CVI showed a negative correlation in MSnON eyes. These associations may reflect ongoing vascular degeneration and possibly inflammatory processes. By contrast, Esen et al reported lower CT in patients with greater disability, suggesting that choroidal alterations in MS could vary with disease stage, phenotype, or methodological differences in imaging and extraction methods.

Unlike most previous studies, we measured CT and CVI across all B-scans from volumetric OCT scan, providing a more comprehensive assessment. Values were averaged across the central circle, inner ring, and OR of the ETDRS grid to capture potential spatial variation. Variability in the literature may be partly attributed to differences in imaging devices, measurement protocols, and confounding factors such as diurnal variation, refractive error, and axial length. We recommend harmonizing imaging and analysis protocols to improve comparability across studies and to better assess the role of choroidal parameters in MS pathophysiology.

Our study has several strenghts. We controlled for key confounders, including IOP and hypertension, by incorporating them into multivariate regression models. Our multimodal imaging approach enabled a comprehensive assessment of both central and peripheral retinal vasculatures, encompassing arterioles, venules, retinal microcapillaries, and choroidal vasculature. For microcapillary density analysis, we included only images with minimal artefacts based on a previously validated classification system^19^ and applied projection artefact removal (see Methods). This rigorous approach helps mitigate significant measurement errors, as emphasized in Murphy et al.^30^ To minimize the impact of factors known to affect CT, such as diurnal variation and lighting conditions, all study visits were consistently scheduled around midday, and imaging was performed under standardized lighting conditions.

Our study has several limitations. The relatively small sample size may have reduced statistical power, potentially explaining why some findings reported in larger cohorts were not replicated. Although linear regression is less sensitive to the number of covariates, there remains a risk of model overfitting when evaluating relationships between RVPs, CVPs, and disability outcomes. Nonetheless, adjusting for key confounders such as age, sex, spherical error, and hypertension was essential, given their known influence on vascular measurements. It is increasingly recognized that different treatments can affect retinal structure;^39^ we did not adjust for treatment type due to our modest sample size, which limited statistical power for subgroup analyses.

## Potential Clinical Relevance

Several RVPs showed statistically significant associations with disability and annual EDSS progression in this exploratory, cross-sectional cohort. In MSnON eyes, increased arteriolar caliber (CRAE: ∼30% increase per EDSS unit), FAZ volume (∼30% per EDSS unit), deep FAZ area (∼10% per EDSS unit), and denser microcapillary networks in the SVC (∼10% per EDSS unit) were associated with faster annual EDSS progression, as were reduced WG (venular WG: ∼10% decrease per EDSS unit) and lower peripheral FD (FD: ∼6% per EDSS unit). Additionally, MS eyes with increased venular caliber (∼3% per EDSS unit), greater VD (∼15% per EDSS unit), denser microcapillary networks (∼4% per EDSS unit), and increased CT (∼5% per EDSS unit), were associated with higher EDSS scores. While these associations are hypothesis-generating and internally consistent, their clinical utility as diagnostic or monitoring biomarkers cannot be established from cross-sectional data in a cohort of this size. Effect sizes were modest, and many changes may lie within the measurement variability of current imaging platforms. These findings should therefore be interpreted as providing preliminary evidence that warrants replication in adequately powered, longitudinal studies before any clinical application is considered.

## Conclusion

In summary, this exploratory study provides evidence that MS is associated with structural alterations in both the retinal and choroidal vasculature, involving not only the microcapillary network but also arterioles, venules, and the peripheral retinal vascular tree, even in eyes without prior ON. These changes may reflect impaired neurovascular coupling and blood–retina barrier dysfunction, potentially secondary to neurodegeneration,^40^ or conversely, driven by hypoperfusion and hypoxia as initiating events.^27^^,^^30^ This interpretation is supported by studies reporting increased arteriolar oxygenation and impaired vascular responses in both the brain and retina.^6^, ^7^, ^8^, ^9^, ^10^ Whether microvascular alterations precede or follow neurodegeneration remains unresolved. The retina offers unique advantages for addressing this question, enabling in vivo, noninvasive assessment of both neural and vascular tissue. However, the present findings are limited by the cross-sectional design and modest sample size, which preclude causal inference and limit statistical power for subgroup analyses. Future studies should adopt longitudinal designs—with larger, well-characterized cohorts, integrating structural and functional measures including oxygenation and vascular reactivity—alongside radiological and inflammatory markers, and accounting for the influence of different disease-modifying therapies. Such studies will be essential for establishing whether retinal and CVPs can serve as validated biomarkers for diagnosis, monitoring, or prognostication in MS.
