# Supplementary material for: Central and Peripheral Alterations of Retinal and Choroidal Vasculature in Multiple Sclerosis: Insights from Multimodal Imaging
Source: Ophthalmol Sci. 2026 Apr 15;6(6):101192. doi: 10.1016/j.xops.2026.101192 (PMC13218244; doi:10.1016/j.xops.2026.101192)
Supplement: Figure S1 [file mmc1.pdf]

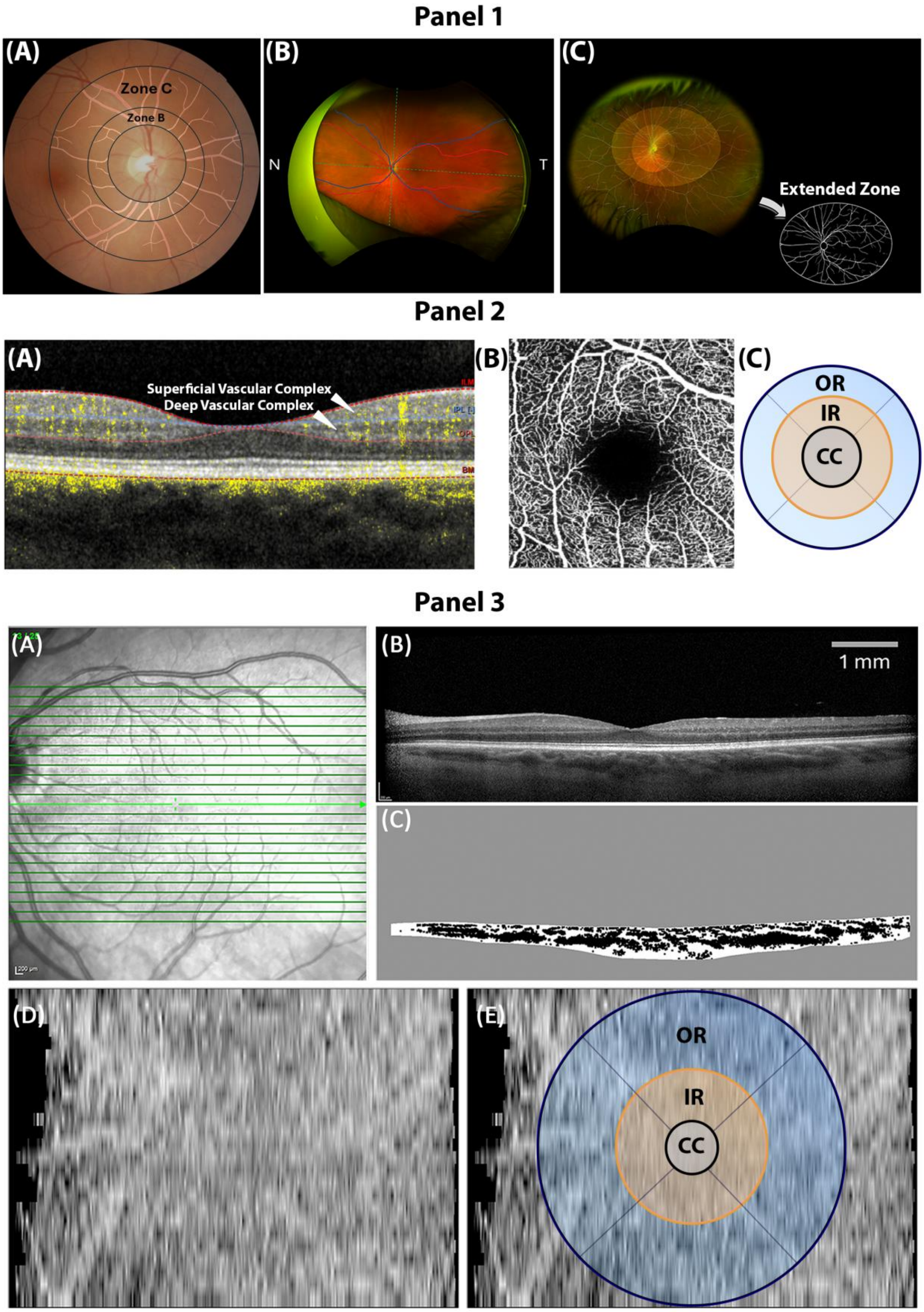

Figure S1. Multimodal Retinal Imaging and Segmentation for Vascular Analysis Across the Central and Peripheral Retina.

Panel A: (A) Fundus image centered on the optic disc showing regions of interest (ROIs) labeled as "zones," with segmented arterioles and venules. (B) Ultra-widefield (UWF) retinal image divided into four quadrants, with major arteries (red) and venules (blue) segmented in each quadrant for width gradient measurements. (C) UWF image illustrating the extended zone used for fractal dimension analysis.

Panel B: Optical Coherence Tomography Angiography (OCTA) with (A) corresponding B-scan showing a foveal cross-section and segmented blood flow (yellow), (B) en face image of the superficial vascular complex vasculature, and (C) ETDRS grid depicting concentric rings used for microcapillary density measurements.

Panel C: (A) Infrared fundus image with green lines indicating the OCT scan area. (B) Representative enhanced-depth imaging (EDI) OCT B-scan of the 25 acquired. (C) Cropped and binarized choroid. (D) Choroidal map generated from all B-scans. (E) Mean choroidal thickness (CT) and choroidal vascularity index (CVI) extracted for each ETDRS grid sector.
