# Supplementary material for: Central and Peripheral Alterations of Retinal and Choroidal Vasculature in Multiple Sclerosis: Insights from Multimodal Imaging
Source: Ophthalmol Sci. 2026 Apr 15;6(6):101192. doi: 10.1016/j.xops.2026.101192 (PMC13218244; doi:10.1016/j.xops.2026.101192)
Supplement: Figure S2 [file mmc2.pdf]

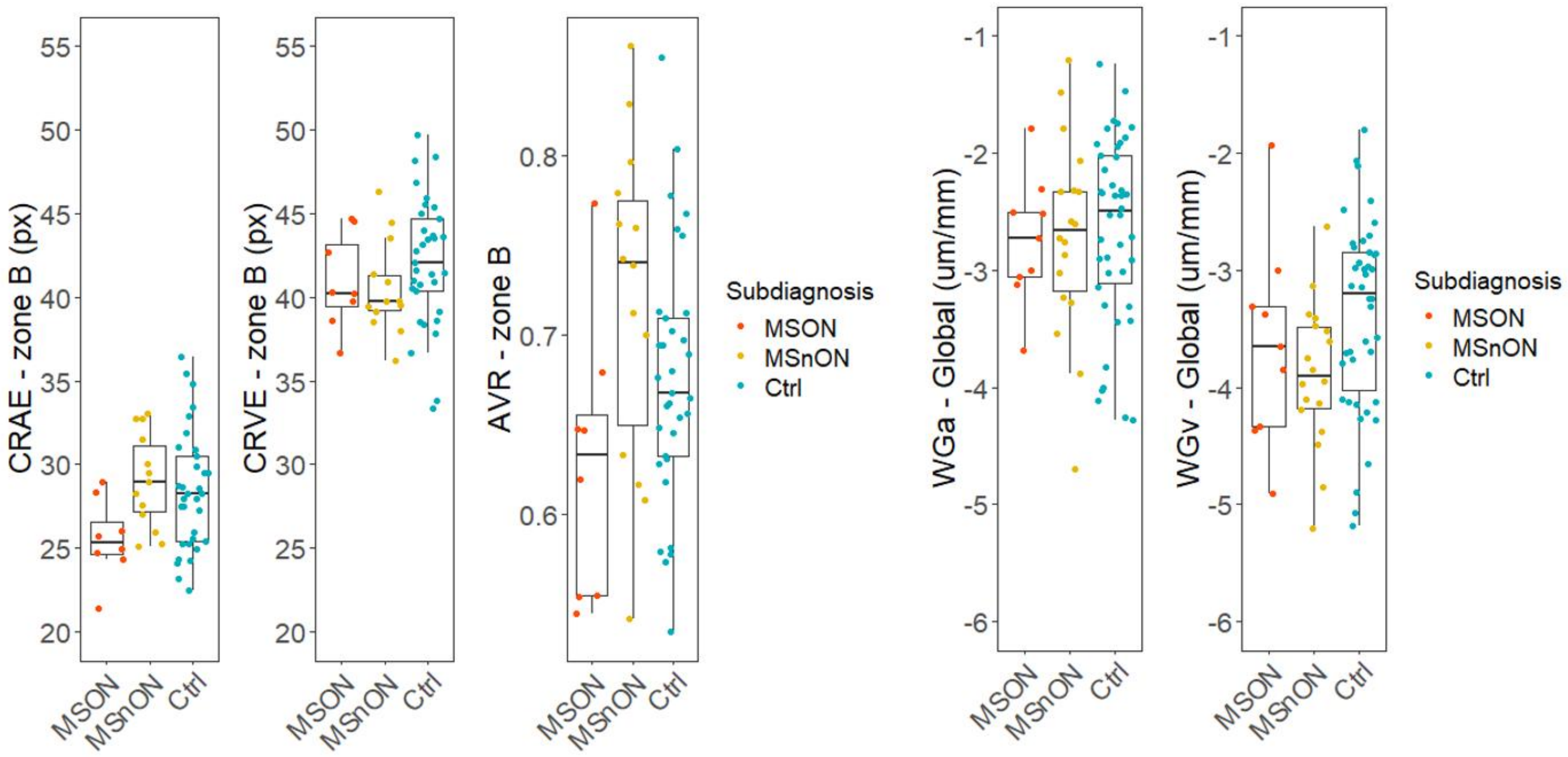

Figure S2. Distribution of Retinal Vessel Calibre Metrics across groups.

Box plots showing retinal vessel calibre metrics: central retinal artery equivalent (CRAE), central retinal vein equivalent (CRVE), arteriole-to-venule ratio (AVR), and width gradient of arteries (WGa) and veins (WGv). Data are shown for eyes from individuals with multiple sclerosis with a history of optic neuritis (MSON; red), without history optic neuritis (MSnON; yellow), and healthy controls (Ctrl; blue). Each data point represents a single eye. CRAE, CRVE, and AVR were measured from zone B using fundus photography, whereas WGa and WGv were derived from ultra-widefield imaging utilising the entire image.
