# Supplementary material for: Central and Peripheral Alterations of Retinal and Choroidal Vasculature in Multiple Sclerosis: Insights from Multimodal Imaging
Source: Ophthalmol Sci. 2026 Apr 15;6(6):101192. doi: 10.1016/j.xops.2026.101192 (PMC13218244; doi:10.1016/j.xops.2026.101192)
Supplement: Figure S3 [file mmc3.pdf]

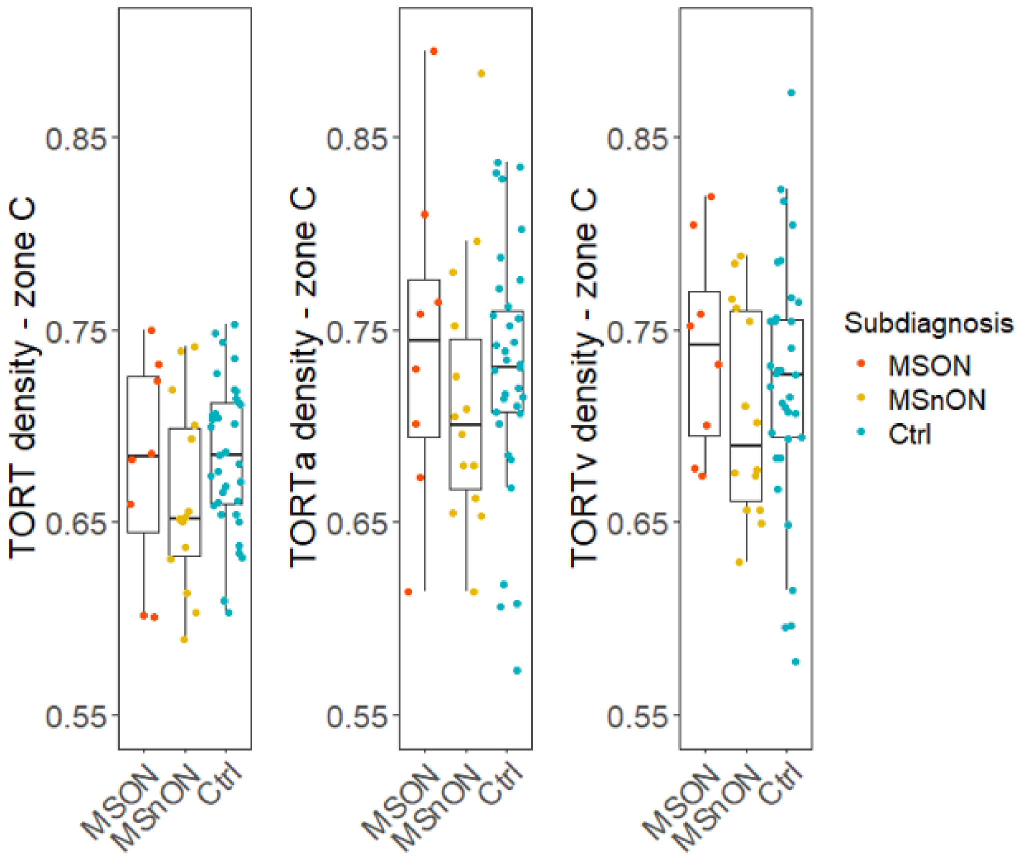

Figure S3. Distribution of Retinal Vessel Tortuosity across groups.

Box plots illustrating tortuosity (TORT) of total retinal vessels, arterioles (a), and venules (v) in eyes from individuals with multiple sclerosis with a history of optic neuritis (MSON; red), without history of optic neuritis (MSnON; yellow), and healthy controls (Ctrl; blue). Each data point represents a single eye. All
