# Supplementary material for: Central and Peripheral Alterations of Retinal and Choroidal Vasculature in Multiple Sclerosis: Insights from Multimodal Imaging
Source: Ophthalmol Sci. 2026 Apr 15;6(6):101192. doi: 10.1016/j.xops.2026.101192 (PMC13218244; doi:10.1016/j.xops.2026.101192)
Supplement: Figure S4 [file mmc4.pdf]

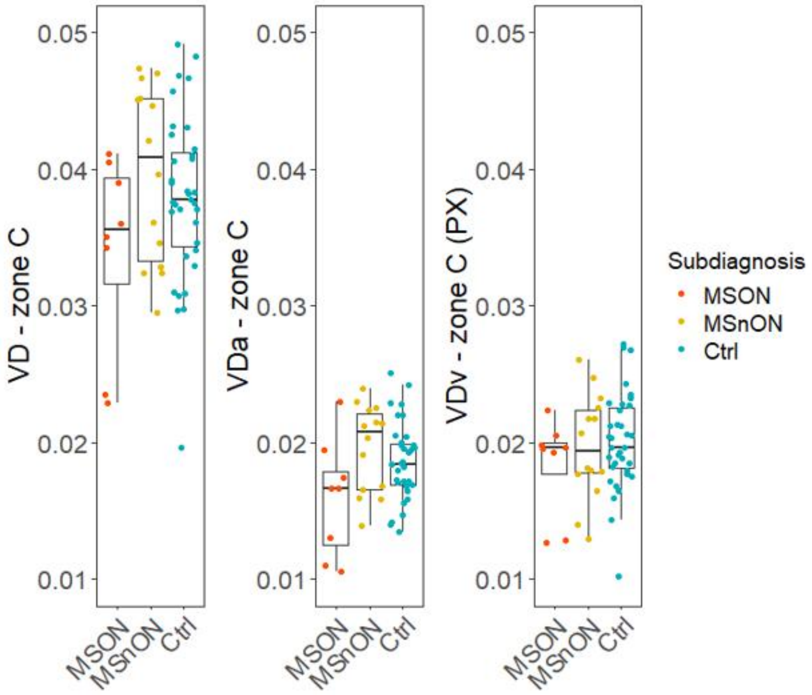

Figure S4. Distribution of Retinal Vessel Density in Multiple Sclerosis across groups.

Box plots illustrating vessel density (VD) of total retinal vessels, arterioles (a), and venules (v) in eyes from individuals with multiple sclerosis with a history of optic neuritis (MSON; red), without history of optic neuritis (MSnON; yellow), and healthy controls (Ctrl; blue). Each data point represents a single eye. All measurements were obtained from zone C using fundus photography.
