# Supplementary material for: Central and Peripheral Alterations of Retinal and Choroidal Vasculature in Multiple Sclerosis: Insights from Multimodal Imaging
Source: Ophthalmol Sci. 2026 Apr 15;6(6):101192. doi: 10.1016/j.xops.2026.101192 (PMC13218244; doi:10.1016/j.xops.2026.101192)
Supplement: Figure S5 [file mmc5.pdf]

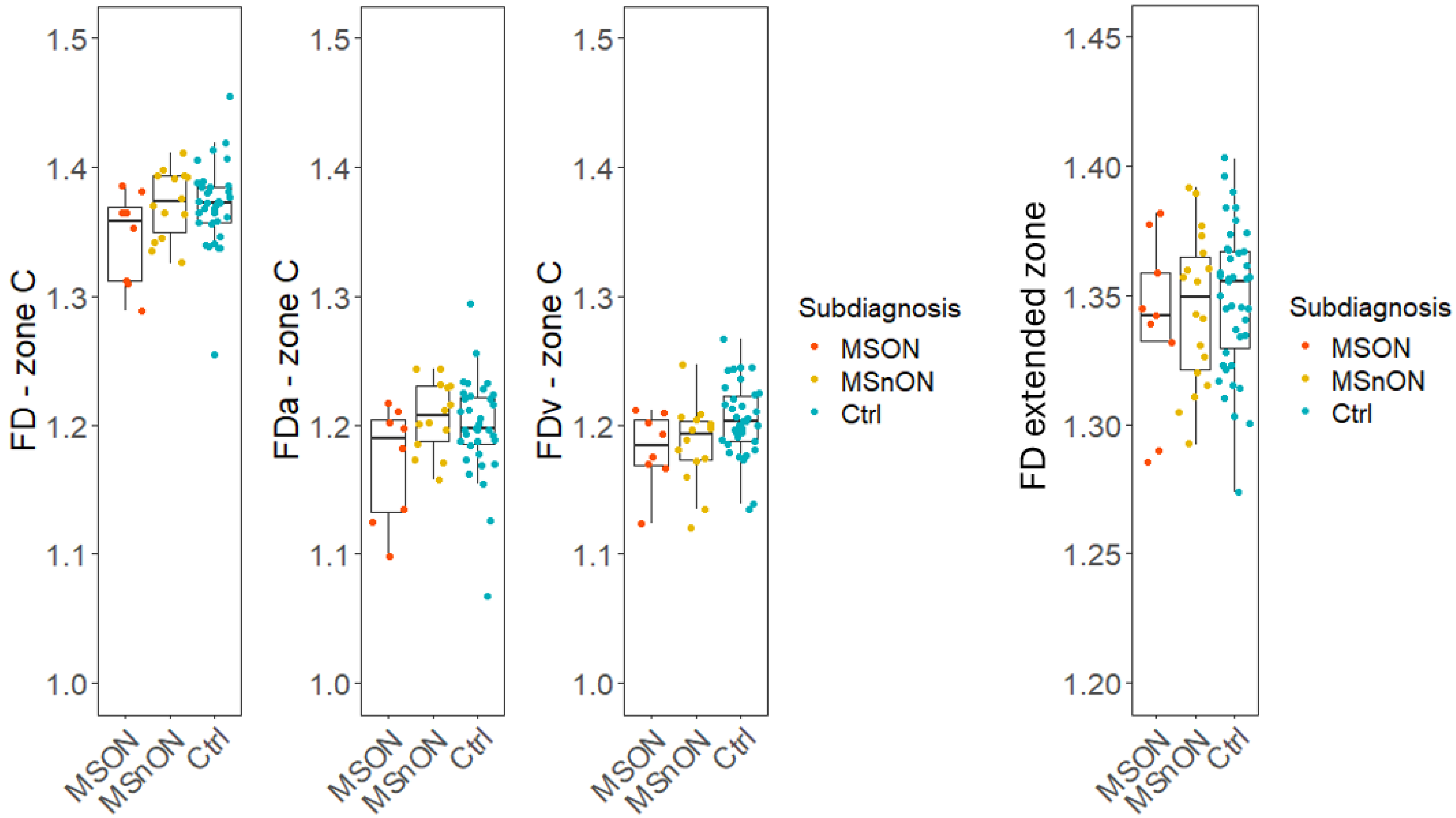

Figure S5. Distribution of Fractal Dimension Retinal Vasculature across groups.

Box plots illustrating fractal dimension (FD) of total retinal vessels, arterioles (FDa), and venules (FDv) in eyes from individuals with multiple sclerosis with a history of optic neuritis (MSON; red), without history of optic neuritis (MSnON; yellow), and healthy controls (Ctrl; blue). Each data point represents a single eye. FD were obtained from zone C using fundus photography and derived from extended zone of the ultra-widefield images.
