# Supplementary material for: Central and Peripheral Alterations of Retinal and Choroidal Vasculature in Multiple Sclerosis: Insights from Multimodal Imaging
Source: Ophthalmol Sci. 2026 Apr 15;6(6):101192. doi: 10.1016/j.xops.2026.101192 (PMC13218244; doi:10.1016/j.xops.2026.101192)
Supplement: Figure S6 [file mmc6.pdf]

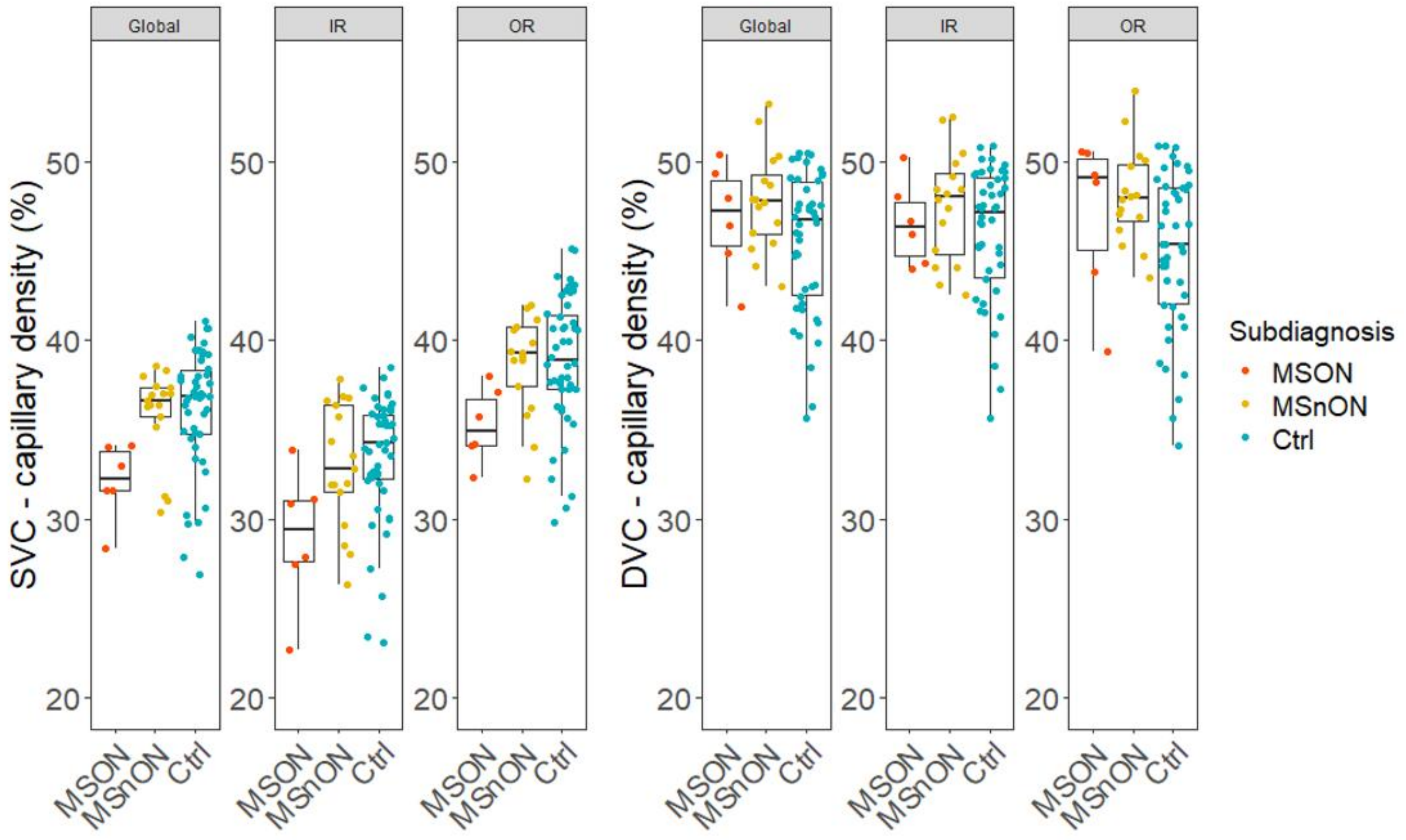

Figure S6. Distribution of Microcapillary Density of Superficial and Deep Vascular Complexes across groups.

Box plots illustrating microcapillary density of the superficial vascular complex (SVC) and deep vascular complex (DVC) in eyes from individuals with multiple sclerosis with a history of optic neuritis (MSON; red), without history of optic neuritis (MSnON; yellow), and healthy controls (Ctrl; blue). Each data point represents a single eye. Measurements were obtained using Optical Coherence Tomography Angiography (OCTA) and recorded for the inner and outer ETDRS rings, as well as for the entire grid (global).
