# Supplementary material for: Central and Peripheral Alterations of Retinal and Choroidal Vasculature in Multiple Sclerosis: Insights from Multimodal Imaging
Source: Ophthalmol Sci. 2026 Apr 15;6(6):101192. doi: 10.1016/j.xops.2026.101192 (PMC13218244; doi:10.1016/j.xops.2026.101192)
Supplement: Figure S7 [file mmc7.pdf]

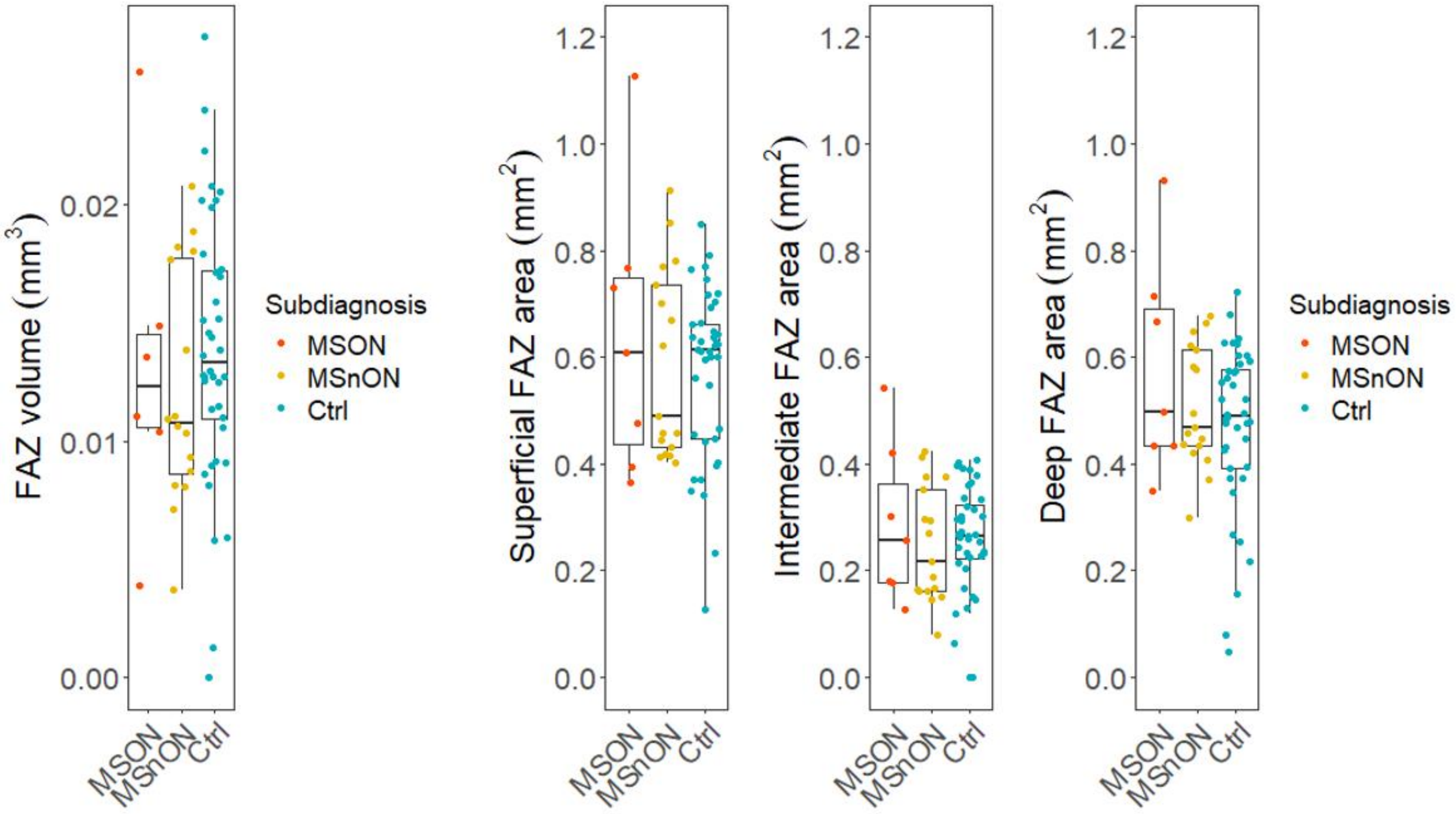

Figure S7. Distribution of Foveal Avascular Zone Metrics across groups.

Box plots illustrating Foveal Avascular Zone (FAZ) volume and area in the superficial, intermediate, and deep vascular complexes in eyes from individuals with multiple sclerosis with a history of optic neuritis (MSON; red), without a history of optic neuritis (MSnON; yellow), and healthy controls (Ctrl; blue). Each data point represents a single eye. All measurements were obtained using Optical Coherence Tomography Angiography (OCTA).
