# Supplementary material for: Central and Peripheral Alterations of Retinal and Choroidal Vasculature in Multiple Sclerosis: Insights from Multimodal Imaging
Source: Ophthalmol Sci. 2026 Apr 15;6(6):101192. doi: 10.1016/j.xops.2026.101192 (PMC13218244; doi:10.1016/j.xops.2026.101192)
Supplement: Figure S8 [file mmc8.pdf]

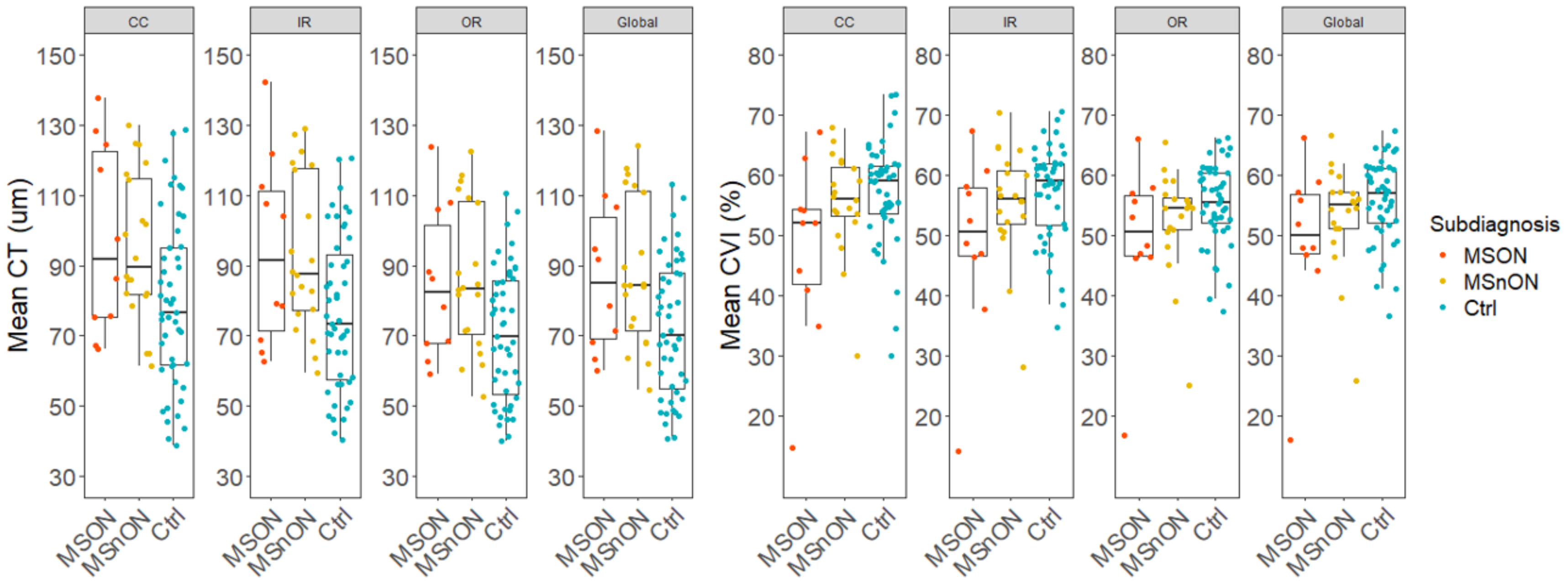

Figure S8. Distribution of Choroidal Thickness and Vascularity across groups.

Box plots illustrating choroidal thickness (CT) and choroidal vascularity index (CVI) in eyes from individuals with multiple sclerosis with a history of optic neuritis (MSON; red), without a history of optic neuritis (MSnON; yellow), and healthy controls (Ctrl; blue). Each data point represents a single eye. All measurements were obtained using Optical Coherence Tomography (OCT) and recorded across multiple ETDRS grid regions: central circle (CC), inner ring (IR), outer ring (OR), and globally (entire grid).
