# Supplementary material for: Central and Peripheral Alterations of Retinal and Choroidal Vasculature in Multiple Sclerosis: Insights from Multimodal Imaging
Source: Ophthalmol Sci. 2026 Apr 15;6(6):101192. doi: 10.1016/j.xops.2026.101192 (PMC13218244; doi:10.1016/j.xops.2026.101192)
Supplement: Table S1 [file mmc9.pdf]

			MSON<br>CFP n=8<br>UWF n=9	MSnON<br>CFP n=14<br>UWF n=18	Ctrl<br>CFP n=33<br>UWF n=42
	**Modality**			**Mean (SD)**	
**Vessel calibre measures**					
CRAE (px)	CFP	zone B	25.5 (2.4)	29.0 (2.7)	28.4 (3.6)
CRVE (px)	CFP	zone B	40.9 (2.8)	40.6 (2.7)	42.1 (3.9)
AVR	CFP	zone B	0.63 (0.08)	0.72 (0.09)	0.68 (0.07)
WGa (um/mm)	UWF	Entire image	-2.75 (0.55)	-2.71 (0.85)	-2.66 (0.80)
WGv (um/mm)	UWF	Entire image	-3.64 (0.88)	-3.89 (0.62)	-3.38 (0.81)
**Vessel tortuosity**					
TORT	CFP	zone C	0.68 (0.06)	0.66 (0.05)	0.68 (0.004)
TORTa	CFP	zone C	0.74 (0.09)	0.71 (0.07)	0.73 (0.06)
TORTv	CFP	zone C	0.74 (0.05)	0.71 (0.05)	0.72 (0.07)
**Vessel density**					
VD	CFP	zone C	0.034 (0.007)	0.040 (0.006)	0.038 (0.006)
VDa	CFP	zone C	0.016 (0.004)	0.02 (0.003)	0.018 (0.003)
VDv	CFP	zone C	0.018 (0.004)	0.02 (0.004)	0.020 (0.004)
**Vascular tree complexity**					
FD	CFP	zone C	1.345 (0.0360	1.372 (0.027)	1.370 (0.032)
FDa	CFP	zone C	1.17 (0.05)	1.206 (0.028)	1.198 (0.038)
FDv	CFP	zone C	1.18 (0.03)	1.19 (0.03)	1.20 (0.03)
FD	UWF	Extended zone	1.339 (0.034)	1.427 (0.014)	1.426 (0.019)

Table S1: Summary of Retinal Vascular Parameters of Arterioles and Venules in MSON, MSnON and Control Eyes. This table presents the mean and standard deviation of key retinal vascular parameters measured from color fundus photographs (CFP) and ultra-widefield (UWF) images across groups: healthy controls (Ctrl), Multiple Sclerosis without optic neuritis (MSnON), and Multiple Sclerosis with optic neuritis (MSON). **Abbreviations:** MSnON, Multiple Sclerosis with no history of optic neuritis; MSON, Multiple Sclerosis with history of optic neuritis; Ctrl, Control; CRAE, Central Retinal Artery Equivalent; CRVE, Central Retinal Vein Equivalent; AVR, Arteriole-to-Venule Ratio; VD, Vessel Density; WG, Width Gradient; TORT, Tortuosity; FD, Fractal Dimension; a, Arteriole; v, Venule; SD, Standard Deviation;
