# Supplementary material for: Central and Peripheral Alterations of Retinal and Choroidal Vasculature in Multiple Sclerosis: Insights from Multimodal Imaging
Source: Ophthalmol Sci. 2026 Apr 15;6(6):101192. doi: 10.1016/j.xops.2026.101192 (PMC13218244; doi:10.1016/j.xops.2026.101192)
Supplement: Table S2 [file mmc10.pdf]

RVPs /location	Modality	MSON (n=6 FAZ:6)	MSnON (n=17 FAZ:16)	Ctrl (n=46 FAZ: n=40)
	**Modality**		**Mean (SD)**	
**SVC capillary density**				
Inner ring	OCTA	28.97 (3.88)	32.99 (3.48)	33.45(3.54)
Outer ring	OCTA	35.23 (2.10)	38.74 (2.76)	38.83 (3.74)
Global	OCTA	32.10 (2.14)	35.86 (2.53)	36.14 (3.42)
**DVC capillary density**				
Inner ring	OCTA	46.54 (2.37)	47.50 (3.09)	46.01 (3.72)
Outer ring	OCTA	47.05 (4.50)	48.09 (2.72)	45.03 (4.37)
Global	OCTA	46.80 (3.12)	47.79 (2.79)	45.52 (3.92)
**FAZ**				
FAZ volume (mm3)	OCTA	0.013 (0.007)	0.012 (0.005)	0.014 (0.006)
SUP FAZ area (µm)	OCTA	0.64 (0.27)	0.59 (0.18)	0.56 (0.18)
INT FAZ area (µm)	OCTA	0.29 (0.15)	0.25 (0.11)	0.26 (0.10)
DEEP FAZ area (µm)	OCTA	0.58 (0.20)	0.51 (0.11)	0.47 (0.16)

Table S2: Summary of Retinal Vascular Parameters of Microcapillary Density in MSON, MSnON and Control Eyes. This table presents the mean and standard deviation of retinal microcapillary metrics measured Optical Coherence Tomography Angiography (OCTA) across groups: healthy controls (Ctrl), Multiple Sclerosis without optic neuritis (MSnON), and Multiple Sclerosis with optic neuritis (MSON). **Abbreviations:** MSnON, Multiple Sclerosis with no history of optic neuritis; MSON, Multiple Sclerosis with history of optic neuritis; Ctrl, Control; SVC, Superficial Vascular Complex; DVC, Deep Vascular Complex; FAZ, Foveal Avascular Zone; SUP, Superior; INT, Intermediate; SD, Standard Deviation;
