# Supplementary material for: Central and Peripheral Alterations of Retinal and Choroidal Vasculature in Multiple Sclerosis: Insights from Multimodal Imaging
Source: Ophthalmol Sci. 2026 Apr 15;6(6):101192. doi: 10.1016/j.xops.2026.101192 (PMC13218244; doi:10.1016/j.xops.2026.101192)
Supplement: Table S3 [file mmc11.pdf]

CVPs /location		MSON (n=10)	MSnON (n=20)	Ctrl (n=47)
	**Modality**		**Mean (SD)**	
**CT (µm)**				
Central circle	OCT-EDI	97.58 (27.28)	94.93 (21.13)	79.42 (24.16)
Inner ring	OCT-EDI	94.28 (27.15)	93.27 (22.21)	76.60 (22.09)
Outer ring	OCT-EDI	84.88 (21.74)	86.24 (20.95)	70.55 (18.77)
Global	OCT-EDI	87.32 (22.74)	88.07 (21.12)	72.15 (19.53)
**CVI (%)**				
Central circle	OCT-EDI	47.79 (15.08)	55.47 (8.48)	57.09 (8.64)
Inner ring	OCT-EDI	49.00 (14.84)	55.41 (9.22)	57.22 (7.96)
Outer ring	OCT-EDI	49.43 (13.13)	52.55 (8.63)	55.25 (6.78)
Global	OCT-EDI	49.30 (13.45)	53.28 (8.66)	55.75 (6.92)

Table S3: Summary of Choroidal Vascular Parameters in MSON, MSnON and Control Eyes. This table presents the mean and standard deviation of choroidal vascular parameters measured from Optical Coherence Tomography (OCT) for each group: healthy controls (Ctrl), Multiple Sclerosis without history optic neuritis (MSnON), and Multiple Sclerosis with history optic neuritis (MSON). **Abbreviations:** MSnON, Multiple Sclerosis with no history of optic neuritis; MSON, Multiple Sclerosis with history of optic neuritis; Ctrl, Control; CT, Choroidal Thickness; CVI, Choroidal Vascularity Index; SD, Standard Deviation;
