# Supplementary material for: Central and Peripheral Alterations of Retinal and Choroidal Vasculature in Multiple Sclerosis: Insights from Multimodal Imaging
Source: Ophthalmol Sci. 2026 Apr 15;6(6):101192. doi: 10.1016/j.xops.2026.101192 (PMC13218244; doi:10.1016/j.xops.2026.101192)
Supplement: Table S4 [file mmc12.pdf]

Variable	N	MSON, N = 8	MSnON, N = 14	p-value[1]
**CRAE**	22			**0.010**
Mean (SD)		25.54 (2.39)	29.02 (2.73)	
Median (IQR)		25.33 (24.60, 26.58)	28.93 (27.15, 31.11)	
Range		21.37, 28.97	25.06, 33.07	
**CRVE**	22			0.664
Mean (SD)		40.92 (2.84)	40.55 (2.68)	
Median (IQR)		40.23 (39.44, 43.14)	39.73 (39.22, 41.26)	
Range		36.65, 44.70	36.23, 46.30	
**AVR**	22			0.059
Mean (SD)		0.63 (0.08)	0.72 (0.09)	
Median (IQR)		0.63 (0.55, 0.66)	0.74 (0.65, 0.77)	
Range		0.54, 0.77	0.54, 0.86	

[1] Wilcoxon rank sum exact test

Variable	N	MSON, N = 9	MSnON, N = 18	p-value[1]
**WGaGlobal**	27			0.860
Mean (SD)		-2.75 (0.55)	-2.71 (0.85)	
Median (IQR)		-2.73 (-3.06, -2.50)	-2.66 (-3.18, -2.32)	
Range		-3.68, -1.79	-4.70, -1.21	
**WGvGlobal**	27			0.463
Mean (SD)		-3.64 (0.88)	-3.89 (0.62)	
Median (IQR)		-3.66 (-4.34, -3.31)	-3.90 (-4.18, -3.49)	
Range		-4.91, -1.94	-5.21, -2.63	

[1] Wilcoxon rank sum exact test

Table S4. Comparison of Retinal Vascular Caliber Metrics across Multiple Sclerosis With and Without a History of Optic Neuritis.

The table presents comparisons of vessel calibre metrics between individuals with multiple sclerosis with a history of optic neuritis (MSON) and those without (MSnON), including mean, median, and range values. **Abbreviations:** MSON, multiple sclerosis with a history of optic neuritis; MSnON, multiple sclerosis with no history of optic neuritis; CRAE, central retinal artery equivalent; CRVE, central retinal vein equivalent; AVR, arteriole-to-venule ratio; WG, width gradient; a, arteriole; v, venule; IQR, interquartile range; SD, standard deviation;
