# Supplementary material for: Central and Peripheral Alterations of Retinal and Choroidal Vasculature in Multiple Sclerosis: Insights from Multimodal Imaging
Source: Ophthalmol Sci. 2026 Apr 15;6(6):101192. doi: 10.1016/j.xops.2026.101192 (PMC13218244; doi:10.1016/j.xops.2026.101192)
Supplement: Table S5 [file mmc13.pdf]

| Variable | N | MSON, N = 8 | MSnON, N = 14 | p-value[1] |
|---|---|---|---|---|
| **TORT zone C** | 22 | | | 0.482 |
| Mean (SD) | | 0.68 (0.06) | 0.66 (0.05) | |
| Median (IQR) | | 0.68 (0.65, 0.73) | 0.65 (0.63, 0.70) | |
| Range | | 0.60, 0.75 | 0.59, 0.74 | |
| **TORTa zone C** | 22 | | | 0.330 |
| Mean (SD) | | 0.74 (0.09) | 0.71 (0.07) | |
| Median (IQR) | | 0.74 (0.69, 0.78) | 0.70 (0.67, 0.75) | |
| Range | | 0.61, 0.90 | 0.61, 0.88 | |
| **TORTv zone C** | 22 | | | 0.238 |
| Mean (SD) | | 0.74 (0.05) | 0.71 (0.05) | |
| Median (IQR) | | 0.74 (0.69, 0.77) | 0.69 (0.66, 0.76) | |
| Range | | 0.67, 0.82 | 0.63, 0.79 | |

[1] Wilcoxon rank sum exact test

| Variable | N | MSON, N = 8 | MSnON, N = 14 | p-value[1] |
|---|---|---|---|---|
| **VD zone C** | 22 | | | 0.145 |
| Mean (SD) | | 0.034 (0.007) | 0.040 (0.006) | |
| Median (IQR) | | 0.036 (0.032, 0.039) | 0.041 (0.033, 0.045) | |
| Range | | 0.023, 0.041 | 0.030, 0.047 | |
| **VDa zone C** | 22 | | | 0.110 |
| Mean (SD) | | 0.016 (0.004) | 0.020 (0.003) | |
| Median (IQR) | | 0.017 (0.013, 0.018) | 0.021 (0.017, 0.022) | |
| Range | | 0.010, 0.023 | 0.014, 0.024 | |
| **VDv zone C** | 22 | | | 0.482 |
| Mean (SD) | | 0.018 (0.004) | 0.020 (0.004) | |
| Median (IQR) | | 0.020 (0.018, 0.020) | 0.019 (0.018, 0.022) | |
| Range | | 0.013, 0.022 | 0.013, 0.026 | |

[1] Wilcoxon rank sum exact test

**Table S5. Comparison of Retinal Vessel Tortuosity and Density in Zone C Across Multiple Sclerosis With and Without a History of Optic Neuritis.**

The table presents comparisons of vessel tortuosity (TORT) and vessel density (VD) between eyes from individuals with multiple sclerosis with a history of optic neuritis (MSON) and those without (MSnON), including mean, median, and range values. Abbreviations: MSON, multiple sclerosis with a history of optic neuritis; MSnON, multiple sclerosis with no history of optic neuritis; TORT, tortuosity; VD, vessel density; a, arteriole; v, venule; IQR, interquartile range; SD, standard deviation;
