# Supplementary material for: Central and Peripheral Alterations of Retinal and Choroidal Vasculature in Multiple Sclerosis: Insights from Multimodal Imaging
Source: Ophthalmol Sci. 2026 Apr 15;6(6):101192. doi: 10.1016/j.xops.2026.101192 (PMC13218244; doi:10.1016/j.xops.2026.101192)
Supplement: Table S6 [file mmc14.pdf]

Variable	N	MSON, N = 8	MSnON, N = 14	p-value[1]
**FD zone C**	22			0.110
Mean (SD)		1.345 (0.036)	1.372 (0.027)	
Median (IQR)		1.359 (1.312, 1.369)	1.374 (1.350, 1.393)	
Range		1.289, 1.386	1.326, 1.411	
**FDa zone C**	22			0.095
Mean (SD)		1.17 (0.05)	1.21 (0.03)	
Median (IQR)		1.19 (1.13, 1.20)	1.21 (1.19, 1.23)	
Range		1.10, 1.22	1.16, 1.24	
**FDv zone C**	22			0.973
Mean (SD)		1.181 (0.029)	1.185 (0.032)	
Median (IQR)		1.184 (1.168, 1.204)	1.193 (1.173, 1.203)	
Range		1.124, 1.212	1.120, 1.247	

[1] Wilcoxon rank sum exact test

Variable	N	MSON, N = 9	MSnON, N = 18	p-value[1]
**FD extended**	27			0.877
Mean (SD)		1.423 (0.018)	1.427 (0.014)	
Median (IQR)		1.428 (1.420, 1.437)	1.428 (1.414, 1.435)	
Range		1.387, 1.440	1.408, 1.453	

Table S6. Comparison of Retinal Vessel Fractal Dimension in Central (zone C) and Extended zone Across Multiple Sclerosis With and Without a History of Optic Neuritis.

The table presents comparisons of fractal dimension (FD) between eyes from individuals with multiple sclerosis with a history of optic neuritis (MSON) and those without (MSnON), including mean, median, and range values. FD in zone C represents measurements in the central retina around the optic disc, while FD in the extended zone reflects values across a broader retinal area. **Abbreviations:** MSON, multiple sclerosis with a history of optic neuritis; MSnON, multiple sclerosis with no history of optic neuritis; FD, fractal dimension; a, arteriole; v, venule; IQR, interquartile range; SD, standard deviation;
