# Supplementary material for: Central and Peripheral Alterations of Retinal and Choroidal Vasculature in Multiple Sclerosis: Insights from Multimodal Imaging
Source: Ophthalmol Sci. 2026 Apr 15;6(6):101192. doi: 10.1016/j.xops.2026.101192 (PMC13218244; doi:10.1016/j.xops.2026.101192)
Supplement: Table S7 [file mmc15.pdf]

SVC (%)	ETDRS Global, N = 23 MSnON N = 17	ETDRS Global, N = 23 MSON N = 6	ETDRS Global, N = 23 p-value[1]	ETDRS IR, N = 23 MSnON N = 17	ETDRS IR, N = 23 MSON N = 6	ETDRS IR, N = 23 p-value[1]	ETDRS OR, N = 23 MSnON N = 17	ETDRS OR, N = 23 MSON N = 6	ETDRS OR, N = 23 p-value[1]
**Capillary density**			**0.010**			**0.030**			**0.013**
Mean (SD)	35.86 (2.53)	32.10 (2.14)		32.99 (3.48)	28.97 (3.88)		38.74 (2.76)	35.23 (2.10)	
Median (Q1, Q3)	36.58 (35.70, 37.34)	32.27 (31.59, 34.03)		32.80 (31.52, 36.41)	29.36 (27.49, 31.11)		39.30 (37.45, 40.71)	34.95 (34.08, 37.06)	
Min, Max	30.39, 38.55	28.36, 34.09		26.30, 37.79	22.64, 33.85		32.25, 41.99	32.34, 38.03	

[1] Wilcoxon rank sum exact test

DVC (%)	ETDRS Global, N = 23 MSnON N = 17	ETDRS Global, N = 23 MSON N = 6	ETDRS Global, N = 23 p-value[1]	ETDRS IR, N = 23 MSnON N = 17	ETDRS IR, N = 23 MSON N = 6	ETDRS IR, N = 23 p-value[1]	ETDRS OR, N = 23 MSnON N = 17	ETDRS OR, N = 23 MSON N = 6	ETDRS OR, N = 23 p-value[1]
**Capillary density**			0.7			0.5			0.9
Mean (SD)	47.79 (2.79)	46.80 (3.12)		47.50 (3.09)	46.54 (2.37)		48.09 (2.72)	47.05 (4.50)	
Median (Q1, Q3)	47.76 (45.76, 49.53)	47.20 (44.87, 49.30)		48.06 (44.54, 49.54)	46.32 (44.32, 48.07)		47.95 (46.53, 49.88)	49.06 (43.79, 50.46)	
Min, Max	43.00, 53.21	41.86, 50.36		42.52, 52.50	43.97, 50.26		43.47, 53.93	39.39, 50.52	

[1] Wilcoxon rank sum exact test

Variable	FAZ volume (mm^3) N	FAZ volume (mm^3) MSON N = 7	FAZ volume (mm^3) MSnON N = 17	FAZ volume (mm^3) p-value[1]	Superficial FAZ area (mm^2) N	Superficial FAZ area (mm^2) MSON N = 7	Superficial FAZ area (mm^2) MSnON N = 17	Superficial FAZ area (mm^2) p-value[1]
**FAZ**	22			0.693	24			>0.999
Mean (SD)		0.013 (0.007)	0.012 (0.005)			0.64 (0.27)	0.59 (0.18)	
Median (Q1, Q3)		0.012 (0.010, 0.015)	0.011 (0.008, 0.018)			0.61 (0.39, 0.77)	0.49 (0.43, 0.74)	
Min, Max		0.004, 0.026	0.004, 0.021			0.37, 1.13	0.40, 0.91	

Variable	Intermediate FAZ area (mm^2) N	Intermediate FAZ area (mm^2) MSON N = 7	Intermediate FAZ area (mm^2) MSnON N = 17	Intermediate FAZ area (mm^2) p-value[1]	Deep FAZ area (mm^2) N	Deep FAZ area (mm^2) MSON N = 7	Deep FAZ area (mm^2) MSnON N = 17	Deep FAZ area (mm^2) p-value[1]
**FAZ**	24			0.576	24			0.534
Mean (SD)		0.29 (0.15)	0.25 (0.11)			0.58 (0.20)	0.51 (0.11)	
Median (Q1, Q3)		0.26 (0.18, 0.42)	0.22 (0.16, 0.35)			0.50 (0.43, 0.72)	0.47 (0.43, 0.61)	
Min, Max		0.13, 0.54	0.08, 0.42			0.35, 0.93	0.30, 0.68	

[1] Wilcoxon rank sum exact test

Table S7. Comparison of Microcapillary Density and Foveal Avascular Zone Metrics Across Multiple Sclerosis With and Without a History of Optic Neuritis.

The table presents comparisons of microcapillary density and Foveal Avascular Zone (FAZ) volume and area between eyes from individuals with multiple sclerosis with a history of optic neuritis (MSON) and those without (MSnON), including mean, median, and range values. FAZ metrics are reported for the superficial, intermediate, and deep vascular complexes, and data are presented across ETDRS grid regions: global (whole grid), inner

ring (IR), and outer ring (OR). **Abbreviations:** MSON, multiple sclerosis with a history of optic neuritis; MSnON, multiple sclerosis with no history of optic neuritis; Ctrl, control; SVC, superficial vascular complex; DVC, deep vascular complex; FAZ, foveal avascular zone; SUP, superior; INT, intermediate; IQR, interquartile range; SD, standard deviation;
