# Supplementary material for: Central and Peripheral Alterations of Retinal and Choroidal Vasculature in Multiple Sclerosis: Insights from Multimodal Imaging
Source: Ophthalmol Sci. 2026 Apr 15;6(6):101192. doi: 10.1016/j.xops.2026.101192 (PMC13218244; doi:10.1016/j.xops.2026.101192)
Supplement: Table S8 [file mmc16.pdf]

Variable	CT CC, N = 30 MSON, N = 10	CT CC, N = 30 MSnON, N = 20	CT CC p-value[1]	CT IR, N = 30 MSON, N = 10	CT IR, N = 30 MSnON, N = 20	CT IR p-value[1]	CT OR, N = 30 MSON, N = 10	CT OR, N = 30 MSnON, N = 20	CT OR p-value[1]	CT Global, N = 30 MSON, N = 10	CT Global, N = 30 MSnON, N = 20	CT Global p-value[1]
**Mean_CT**			0.8			0.9			0.8			0.8
Mean (SD)	97.58 (27.28)	94.93 (21.13)		94.28 (27.15)	93.27 (22.21)		84.88 (21.74)	86.24 (20.95)		87.32 (22.74)	88.07 (21.12)	
Median (IQR)	91.87 (75.36, 122.67)	89.44 (81.83, 114.87)		91.57 (71.35, 111.30)	87.74 (77.30, 117.59)		82.32 (67.97, 101.57)	83.25 (70.40, 108.23)		85.15 (69.01, 103.75)	84.35 (71.47, 111.22)	
Range	66.30, 137.77	61.31, 130.00		62.61, 142.23	59.35, 129.06		59.27, 123.73	52.77, 122.67		60.24, 128.25	54.54, 124.21	

[1] Wilcoxon rank sum exact test

Variable	CVI CC, N = 30 MSON, N = 10	CVI CC, N = 30 MSnON, N = 20	CVI CC p-value[1]	CVI IR, N = 30 MSON, N = 10	CVI IR, N = 30 MSnON, N = 20	CVI IR p-value[2]	CVI OR, N = 30 MSON, N = 10	CVI OR, N = 30 MSnON, N = 20	CVI OR p-value[2]	CVI Global, N = 30 MSON, N = 10	CVI Global, N = 30 MSnON, N = 20	CVI Global p-value[2]
**Mean_CVI**			0.12			0.2			0.5			0.4
Mean (SD)	47.79 (15.08)	55.47 (8.48)		49.00 (14.84)	55.41 (9.22)		49.43 (13.13)	52.55 (8.63)		49.30 (13.45)	53.28 (8.66)	
Median (IQR)	52.15 (41.80, 54.35)	56.10 (53.28, 61.33)		50.60 (46.63, 57.85)	56.05 (51.98, 60.80)		50.65 (46.58, 56.60)	54.50 (50.90, 56.20)		49.95 (47.08, 56.80)	55.05 (51.20, 57.13)	
Range	14.70, 67.20	30.00, 67.80		14.20, 67.40	28.20, 70.30		16.70, 66.00	25.00, 65.50		16.10, 66.30	25.90, 66.60	

[1] Wilcoxon rank sum exact test

[2] Wilcoxon rank sum test

Table S8. Comparison of Choroidal Thickness and Vascularity Index Across Multiple Sclerosis With and Without a History of Optic Neuritis.

The table presents comparisons of choroidal thickness (CT) and choroidal vascularity index (CVI) between eyes from individuals with multiple sclerosis with a history of optic neuritis (MSON) and those without (MSnON), including mean, median, and range values. Measurements were recorded across regions defined by the ETDRS grid: central circle (CC), inner ring (IR), outer ring (OR), and globally (whole grid). Abbreviations: MSON, multiple sclerosis with a history of optic neuritis; MSnON, multiple sclerosis with no history of optic neuritis; Ctrl, control; CT, choroidal thickness; CVI, choroidal vascularity index; IQR, interquartile range; SD, standard deviation;
